# Metaproteome Analysis of Short‐Term Thermal Stress in Three Sympatric Coral Species Reveals Divergent Host Responses

**DOI:** 10.1002/ece3.73275

**Published:** 2026-03-19

**Authors:** Shrinivas Nandi, Timothy G. Stephens, Erin E. Chille, Samantha Goyen, Line K. Bay, Debashish Bhattacharya

**Affiliations:** ^1^ Department of Biochemistry and Microbiology Rutgers University New Brunswick New Jersey USA; ^2^ Australian Institute of Marine Science Townsville Queensland Australia

**Keywords:** coral bleaching, great barrier reef, holobiont, metaproteomics, protein abundance, stony corals, thermal stress

## Abstract

Anthropogenic climate change has contributed to the accelerating loss of coral reefs worldwide. This crisis has led to a myriad of studies aimed at understanding the basis of coral resilience to support reef conservation. Here, we compare physiological, proteomic, and metabolomic responses to acute thermal stress to identify both diverged and conserved stress response strategies and molecular markers of bleaching susceptibility in three different coral species. We find species‐specific responses with the thermally sensitive *Acropora hyacinthus* exhibiting a rapid decline in endosymbiont physiology (~19% decline in photosynthetic efficiency and a −1.88 fold change in abundance), coupled with one‐third of proteins showing a reduction in abundance. In contrast, *Porites lobata* displayed a delayed physiological and proteomic (~5% initial; ~14% prolonged) response to stress, suggesting greater resilience. *Stylophora pistillata* initially showed shifts in the proteome (~11%) followed by colony “bail‐out”, that is, rapid tissue loss. Overall, we observed markedly different responses in most biochemical pathways in the three coral species. Nonetheless, some known biomarkers of stress, including heat‐shock proteins, showed conserved, cross‐species responses to thermal stress with differences in temporal abundance reflecting bleaching resistance. Metabolomic profiling revealed an increase in stress‐associated dipeptides and free amino acids in all three species, although species‐specific and temporally variable responses occurred. Our results underscore the species‐specific nature of coral responses to thermal stress and highlight proteomic signatures associated with symbiosis breakdown, offering mechanistic insights into coral bleaching susceptibility and resilience. Overall, these findings enhance our ability to identify early‐warning indicators of bleaching and underscore the challenges associated with the development of universal coral stress biomarkers.

## Graphical abstract

Short‐term thermal stress triggers distinct molecular responses in three sympatric coral species with contrasting thermal resilience. Proteomic and metabolomic profiling reveal both species‐specific and limited shared pathways underlying these stress responses. These findings highlight the complexity of coral bleaching mechanisms and the challenges in developing universal diagnostic biomarkers.

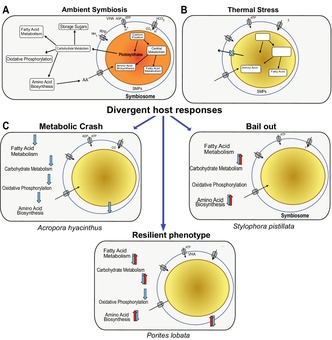

## 1 Introduction

Stony corals (Scleractinia) thrive in oligotrophic waters due to their ability to form a symbiotic association with dinoflagellate algae in the family Symbiodiniaceae which provide the host with photosynthates (Gordon and Leggat 2010; Muscatine and Porter 1977). In exchange, corals provide Symbiodiniaceae with a stable, sheltered environment, inorganic carbon, and other essential nutrients (Frankowiak et al. 2016). Symbionts reside within gastrodermal symbiosome compartments that optimize intracellular conditions for photosynthesis (Barott et al. 2022, 2015; Bertucci et al. 2013; Thies et al. 2022). Under stressful conditions (e.g., high nutrient enrichment, temperature stress), coral hosts may lose their algal symbionts, which is known as coral bleaching. During bleaching, some species can sustain themselves for extended periods of time through heterotrophic feeding (Grottoli et al. 2006), but bleached corals remain more susceptible to disease and mortality (Bourne et al. 2016; Muller et al. 2008). The increase in mean global oceanic temperature, a result of anthropogenic climate change, has led to increased bleaching episodes and a significant decline in global coral populations (Hughes et al. 2017).

Although coral bleaching is a broadly conserved stress response across Scleractinia, susceptibility to temperature fluctuations varies widely among species due to ecological, physiological, and evolutionary factors (Loya et al. 2001; van Woesik et al. 2011). On the Great Barrier Reef, bleaching outcomes have been extensively documented (Burn et al. 2023; Hughes et al. 2019, 2018, 2017; Pratchett et al. 2020), with massive, slow‐growing corals such as *Porites* generally exhibiting greater thermal tolerance than branching, faster‐growing genera such as *Acropora* (Burn et al. 2023; Fitt et al. 2009; Marshall and Baird 2000; Pratchett et al. 2020), likely reflecting contrasting energy‐allocation strategies. In addition, traits including skeletal morphology (Swain et al. 2018, 2016), tissue thickness (Barkley et al. 2018; Qin et al. 2020), heterotrophic plasticity (Grottoli et al. 2006; Martinez et al. 2024; Sangmanee et al. 2020), resident algal symbionts (Bhattacharya et al. 2024; Buerger et al. 2020; Putnam et al. 2017; Rowan 2004; Wang et al. 2023; Ziegler et al. 2018), and composition of the prokaryotic microbiome (Peixoto et al. 2017; Reshef et al. 2006; Rosado et al. 2019; Ziegler et al. 2017) also influence thermal tolerance. Local environmental conditions such as water flow, light, nutrient levels, and prior exposure to sublethal or variable temperatures can further modulate bleaching severity (Barshis et al. 2013; Kenkel et al. 2013; Morgan et al. 2017; Oliver and Palumbi 2011; Risk and Edinger 2011; Safaie et al. 2018).

Developing reliable molecular biomarkers to detect early stress responses in corals is a major goal in reef monitoring and conservation. These tools will enable rapid, pre‐bleaching health assessment, which can guide targeted interventions (Chille et al. 2025; Downs et al. 2005). Transcriptomic datasets have been extensively used to identify putative biomarkers of thermal stress (Chille et al. 2025; Cowen and Putnam 2022; Cziesielski et al. 2019). However, a central challenge is that stress produces high variation in gene expression levels in different species (Da‐Anoy et al. 2024; Ip et al. 2022; Rosic et al. 2020), and even among genotypes within a species (Chille et al. 2024; Kenkel and Matz 2016). Nonetheless, transcriptomic studies have identified some marker genes of thermal stress, including those encoding heat‐shock proteins (e.g., HSP70, HSP90) (Nakamura et al. 2011; Rosic et al. 2011; Seveso et al. 2016), ubiquitin‐related proteins (DeSalvo et al. 2008; Rodriguez‐Lanetty et al. 2009; Seneca and Palumbi 2015), and antioxidant enzymes (Downs et al. 2002; Krueger et al. 2015) among others. A recent review of 307 gene and protein expression papers studying stress response in cnidarians found that most genes have inconsistent responses across different experiments, with only 14 having shared responses across at least two species tested and NF‐κB having shared responses across four species, thereby suggesting a high degree of species specificity vis‐à‐vis the coral stress response (Molinari et al. 2025). This high variability in transcriptional responses underscores the need for proteomic approaches, which directly measure the functional molecules involved in stress responses and may therefore identify more broadly applicable biomarkers (Csárdi et al. 2015). Proteomics also captures post‐transcriptional regulation and protein modification that transcriptomics alone cannot resolve (Greenbaum et al. 2003), providing a mechanistic link between gene expression and cellular phenotype. Furthermore, proteins have practical diagnostic relevance because they are more easily incorporated into point‐of‐care detection tools (Chille et al. 2025). In addition, work in diverse eukaryotes, including the coral *Montipora capitata*, shows that transcript abundance often correlates poorly with protein levels (Williams et al. 2023), further supporting the use of proteomics as a complementary and necessary tool for biomarker discovery and for elucidating coral thermal stress mechanisms (Chille et al. 2025).

Metabolomics provides complementary insights to proteomic data by capturing the end products of cellular activity, that is, metabolites that directly reflect the real‐time physiological state of the organism (Fiehn 2002; Roessner and Bowne 2009). Whereas proteomics identifies the functional proteins associated with stress, metabolomics reveals the downstream biochemical consequences of those protein‐level changes, making the two approaches complementary for resolving both mechanism and phenotype in coral stress biology (Johnson et al. 2016). Previous metabolomic studies in corals have reported changes in antioxidant‐related metabolites during thermal stress (Williams, Chiles, et al. 2021) and shifts in amino acid pools (Chiles et al. 2022; Martinez, Grover, Baker, and Ferrier‐Pagès 2022; Martinez et al. 2020). Notably, recent findings have demonstrated the accumulation of specific dipeptides as reliable indicators of pre‐bleaching thermal stress. These include lysine‐glutamine (KQ), arginine‐glutamine (RQ), arginine‐alanine (RA), and arginine‐valine (RV) (Huffmyer et al. 2024; Williams, Pathmanathan, et al. 2021).

**FIGURE 1:**
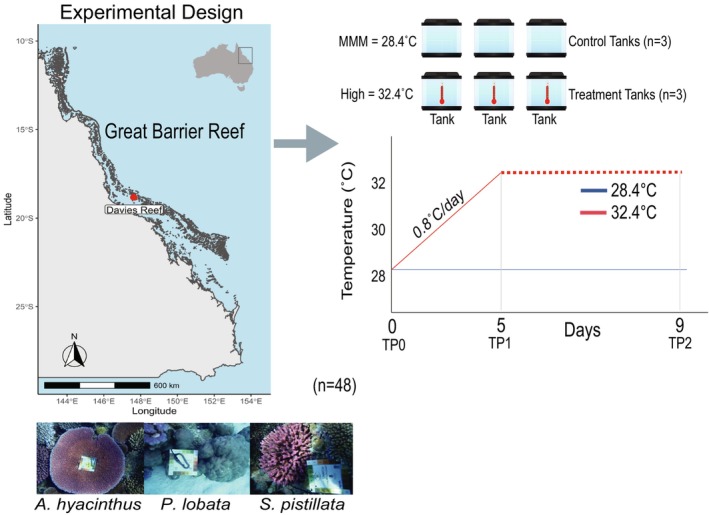
Study sites and experimental designs. Location of samples (*n* = 48) collected and the experimental design (right panel) are shown. The coral images were generated by S. Goyen. TP refers to the timepoint at which corals were sampled. Tank set up is displayed on the right with the mean temperatures of each tank shown. Graph showing the ramp up protocol used for the tanks, red, thermal stress tanks and blue, control tanks.

In this study, we used proteomic data to investigate the coral holobiont stress response in three sympatric species from the Great Barrier Reef (GBR), Australia: *Acropora hyacinthus* , *Porites lobata* , and *Stylophora pistillata* (Figure 1). These species were selected because they represent major reef‐building corals on the GBR and span distinct ecological, morphological, and physiological strategies, including differences in thermal sensitivity ranging from sensitive to resilient (Burn et al. 2023; Fitt et al. 2009; Manalili et al. 2025; Marshall and Baird 2000; Pratchett et al. 2020). Here, we asked how both proteomic and metabolomic responses to acute heat stress diverge among species with contrasting thermal sensitivities, and which components of these responses are conserved across all three taxa. To address these questions, we examined key metabolic pathways in each species to better understand their responses to thermal stress and searched for conserved proteins across species that can potentially be used as biomarkers. Lastly, we investigated the metabolomic data to validate previously reported shifts in metabolite abundance, including dipeptides, amino acids, and antioxidants that play key roles in the coral thermal stress response.

## 2 Results

### 2.1 Host Bailout and Mortality

Briefly, coral fragments were acclimated at ~28.4°C and then subjected to a heat treatment ramping to 32.4°C over 5 days (0.8°C/day). Samples were collected at three timepoints: before ramp‐up (day 0, TP0), a the end of ramp up (day 5, TP1) and after 4 days at maximum temperature (day 9, TP2) (Figure 1). No mortality was observed in *A. hyacinthus* and *P. lobata* . In contrast, *S. pistillata* exhibited significant tissue loss (i.e., “bail‐out”) and loss of color (becoming paler) based on visual assessment at TP1 (Figure S1), with complete tissue loss observed by TP2 for most fragments.

### 2.2 Endosymbiont Community Composition and Physiology

**FIGURE 2:**
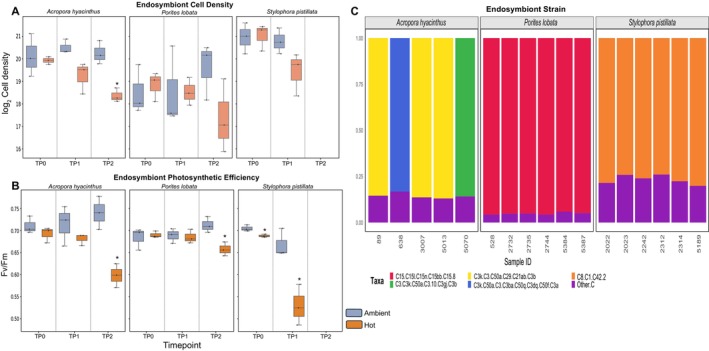
Physiological measurements of GBR corals under ambient and heat stress treatments. (A) Endosymbiont cell abundance (log_2_‐transformed) across the experimental duration under ambient and thermal stress conditions for each coral species. (B) Maximum quantum yield of photosystem II (*F*_*v*_/*F*_*m*_) measured across experimental time points under ambient and thermal stress conditions; asterisks indicate significant differences between treatments at a given time point (*p* < 0.05). (C) Relative abundance of dominant Symbiodiniaceae ITS2 profiles (SymPortal) associated with *Acropora hyacinthus* , *Porites lobata* , and *Stylophora pistillata* colonies from the Great Barrier Reef.

Because coral bleaching is associated with reduced algal symbiont abundance in host tissues and reduced photosynthetic efficiency, cell counts (Figure 2A) and Pulse Amplitude Modulation (PAM) fluorometer measurements (Figure 2B) were taken for each of the samples, collected at each time point to characterize the effect of thermal stress on endosymbiont physiology in the three coral species (Table S1). Symbiont cell densities, calculated as a factor of coral tissue area; hereinafter, endosymbiont abundance, were used to assess bleaching severity and to normalize the proteomic data generated from the symbionts in each of the sampled coral fragments. The latter was done to account for reduced symbiont protein abundance values because of symbiont cell loss and not reduced accumulation of the protein within the symbiont cells. Under ambient conditions, endosymbiont abundances remained stable across the experiment (Figure 2A). Prior to ANOVA, model assumptions were evaluated for each species independently, including normality (Shapiro–Wilk test, *p* > 0.05) and variance (Levene's test, *p* > 0.05, Data S1). Using ANOVA, we observed significant shifts in endosymbiont abundance upon exposure to thermal stress in *A. hyacinthus* (df = 5, *F* = 6.032, *p*‐value = 0.0051), but not in *P. lobata* (df = 5, *F* = 1.015, *p*‐value = 0.450) or *S. pistillata* (df = 3, *F* = 3.285, *p*‐value = 0.079). In *A. hyacinthus* , we observed significant declines in endosymbiont counts over the experimental timepoint comparing the FC between ambient and thermal stress samples (TP0 = −0.19, TP1 = −1.26 and TP2 = −1.88). Tukey‐HSD post hoc analysis indicated a significant difference only at TP2 (adj‐*p‐value* = 0.014). In *P. lobata* , endosymbiont abundance showed little difference between ambient and thermal treatments at TP0 (FC = 0.34) and TP1 (FC = −0.01) but declined sharply under thermal stress by TP2 (FC = −2.26), though the values were not statistically significant. Likewise, in *S. pistillata* we observed minimal shifts at TP0 (FC = 0.08) and TP1 (FC = −1.35).

Same as endosymbiont abundance, photosynthetic efficiency (*F*_*v*_ */F*_*m*_) values remained stable under ambient conditions in all three species. However, under thermal stress, we observed significant declines in endosymbiont photosynthetic efficiency in all three coral species (Figure 2B). ANOVA models were verified for normality and variance (see Data S1). ANOVA analysis showed significant photosynthetic efficiency shifts in *A. hyacinthus* (df = 5, *F* = 8.552, *p*‐value = 0.0011). Tukey‐HSD post hoc analyses revealed a significant decrease in thermal stress samples at TP2 (adj‐*p*‐value = 0.0007, 19.16% decrease under stress). Similarly, in *P. lobata* (ANOVA: df = 5.0, *F* = 3.067, *p‐*value = 0.051), a significant decline was observed only at TP2 (adj‐*p*‐value = 0.020, 7.65% decrease under stress). Lastly, in *S. pistillata* (df = 5, *F* = 23.89, *p*‐value = 0.00024), we observed a significant decline at TP1 (adj‐*p‐*value = 0.0015, 20.70% decline under stress).

We also characterized *Symbiodiniaceae* endosymbiont community profiles using ITS2 sequencing (Figure 2C) to determine whether differences in host performance could be attributed to differences in resident endosymbiont composition. ITS2 sequencing yielded 1.54 million total Symbiodiniaceae sequences, with an average post‐MED read depth of 86,000 reads per sample ( *A. hyacinthus* 43,447 reads; *P. lobata* 109,069 reads; *S. pistillata* 104,196 reads). Pre‐MED sequencing depth averaged 88,350 reads per sample, with an overall retention fraction of 96.6% after MED filtering (post‐MED/pre‐MED). Across species, mean retention fractions were 0.95 ( *A. hyacinthus* ), 0.98 ( *P. lobata* ), and 0.96 ( *S. pistillata* ). One *A. hyacinthus* sample (5016_WOO13321A243) yielded only 96 reads and was excluded from all downstream analyses. The sample is shown in the relative abundance plot (Figure 2C) for completeness but was not included in any statistical or comparative analyses. All other samples exceeded accepted read‐depth thresholds for ITS2 profiling. Each sample represents a single biological replicate processed once in SymPortal. This analysis showed that all samples associated exclusively with the genus *Cladocopium* (except for one *P. lobata* replicate, which had < 1% relative abundance of *Gerakladium*) (Figure 2C). The three coral species exhibited distinct major ITS2 type profiles, which were C15‐C15l‐C15n‐C15bb‐C15.8 for all samples of *P. lobata* , C8/C1‐C42.2 for all samples of *S. pistillata* , and C3k/C3‐C50a‐C29‐C21ab‐C3b, C3/C3k‐C50a‐C3.10‐C3gj‐C3b, and C3k‐C50a‐C3‐C3ba‐C50q‐C3dq‐C50f‐C3a for all samples of *A. hyacinthus* (Table S1b).

### 2.3 Tank Effects in the Endosymbiont Proteome

After quality filtering of endosymbiont protein groups, we obtained 332, 275, and 184 proteins from the endosymbionts of *A. hyacinthus* , *P. lobata* , and *S. pistillata* , respectively. Protein abundances were normalized by endosymbiont cell density to account for changes in abundance across samples, thereby minimizing artifacts caused by bleaching‐induced cell loss. PCA analysis, along with assessments of median protein intensities (File S2), revealed strong tank effects that led to proteomic shifts even among ambient samples, independent of thermal stress. These confounding effects limited our ability to interpret biological signal; thus, we did not pursue further analysis of the endosymbiont proteome.

### 2.4 Overall Host Proteome Analysis

Prior to filtering, we obtained 3793 protein groups (hereinafter proteins) from *A. hyacinthus* (Table S2), 4098 proteins from *P. lobata* (Table S3), and 2479 proteins from *S. pistillata* (Table S4). After filtering for unique and razor peptides and removing proteins with low counts, 2255 ( *A. hyacinthus* ), 2351 ( *P. lobata* ), and 1335 ( *S. pistillata* ) proteins remained for downstream analysis. We assessed differential abundance for all proteins using pairwise comparisons. A protein was considered to have increased abundance if it had a log_2_ fold‐change (FC) > 0.5 and *p*‐value < 0.05, and decreased abundance if it had a FC < −0.5 with *p*‐value < 0.05.

Differentially abundant proteins were detected at TP0 in all three species, suggesting the presence of tank effects. In *A. hyacinthus* , 72 proteins showed increased abundance and 5 showed decreased abundance (Figure S2). In *P. lobata* , 46 proteins had increased abundance and 82 had decreased abundance. In *S. pistillata* , 5 proteins exhibited increased abundance and 148 exhibited decreased abundance. Although these tank effects at TP0 represent an experimental limitation, the larger proteomic shifts observed at TP1 and TP2 suggest that they are unlikely to substantially influence downstream thermal‐stress comparisons. In *A. hyacinthus* at TP1, 23 proteins showed increased abundance and 161 showed decreased abundance; at TP2, 75 proteins showed increased abundance and 101 showed decreased abundance. In *P. lobata* at TP1, 23 proteins showed increased abundance and 96 showed decreased abundance; at TP2, 86 proteins showed increased abundance and 235 showed decreased abundance (Figure S2). Lastly, in *S. pistillata* , 87 proteins showed increased abundance and 57 showed decreased abundance at TP1. Samples were collected only for two timepoints, due to complete bailout by TP2 (see 3. Discussion). These temporal patterns also suggest species‐specific proteomic responses, with *A. hyacinthus* and *S. pistillata* exhibiting larger early shifts in protein abundance compared to the more gradual response observed in *P. lobata* .

### 2.5 Pathway Analysis of Coral Hosts

**FIGURE 3:**
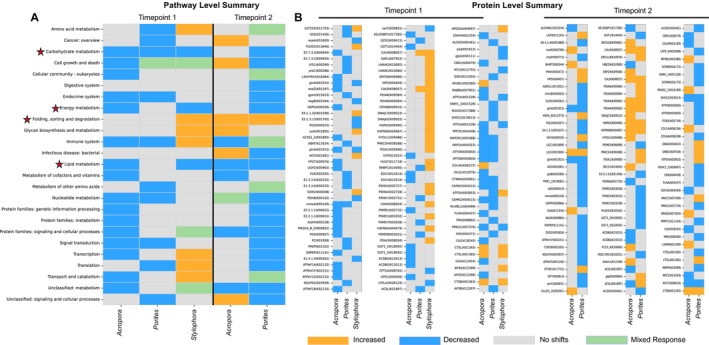
Proteomic responses at the pathway level and individual protein abundances in conserved pathways. (A) Heatmap summarizing pathway‐level responses across coral species and time points. Pathways are shown on the y‐axis and species on the x‐axis; colors denote response categories as defined in the figure key. Red stars highlight conserved pathways discussed in the main text. (B) Heatmap of significantly responding proteins (|FC| > 0.5, *p*‐value < 0.05) across species and time points, with proteins (name and KEGG ID) shown on the y‐axis. Panels are separated by time point (TP1, left; TP2, right).

Enrichment analysis at the “B‐level” (KEGG Orthology) was done to compare pathway‐level differences in coral stress responses. Traditional tools for pathway level enrichment such as Gene Set Enrichment Analysis (GSEA) require abundance data for most genes in a pathway to allow robust statistical inferences. However, an observed limitation in proteomic data is the low coverage of the proteome: only ~9.7% of *A. hyacinthus* proteins were detected in the proteomic data, only ~5.1% of *S. pistillata* proteins, and 11.1% of *P. lobata* proteins. We therefore utilized a percentage‐based metric to assess pathway level response (Oakley et al. 2017; Petrou et al. 2021), that is, pathways were considered upregulated if ≥ 5% of the total proteins in a pathway showed increased abundance under stress, and downregulated if ≥ 5% showed a decrease. The 5% value was chosen to serve as a lenient cut‐off, thereby allowing us to detect subtle, coordinated pathway responses while reducing the influence of single‐protein outliers. Mixed responses were assigned when both patterns were observed. After filtering for pathways with more than 10 proteins in each species, 25 pathways were retained for analysis (Figure 3A). At TP1, *A. hyacinthus* showed downregulation in 16 pathways; *P. lobata* showed downregulation in 10 pathways and a mixed response in one; *S. pistillata* showed upregulation in seven pathways, downregulation in three, and a mixed response in three. By TP2, *A. hyacinthus* displayed a shift, with 5 pathways downregulated, six upregulated, and one mixed. In contrast, *P. lobata* exhibited stronger downregulation at TP2, with 16 pathways downregulated, five mixed, and one upregulated. Several pathways downregulated in *P. lobata* at TP2 mirrored those observed in *A. hyacinthus* at TP1 (e.g., energy metabolism).

To identify shared vs. species‐specific responses, we examined conserved patterns, defined as a consistent direction of regulation across all species, and divergent responses (i.e., opposing responses between species). Pathways were considered conserved even if the trend emerged at different timepoints. Such temporal differences were consistent with species‐specific progression of thermal stress, with more sensitive corals showing earlier pathway shifts than more resilient species. For example, energy metabolism was downregulated at TP1 in both *S. pistillata* and *A. hyacinthus* , but in *P. lobata* , this downregulation occurred only at TP2. This approach accounts for differences in bleaching rates among the species, as evident from physiological measurements. Only four pathways showed conserved response wherein carbohydrate metabolism, energy metabolism, lipid metabolism were downregulated, and folding/sorting/degradation was upregulated (Figure 3A, red stars). In contrast, 21 pathways displayed divergent responses (for example: cell growth and death) or species‐specific responses (for example: downregulation of “digestive system” in *P. lobata* only).

We prioritized conserved pathways for additional functional analysis. Although “amino acid metabolism” showed divergent trends, it was included due to its known role in thermal stress in corals. Furthermore, two custom sets of proteins, symbiosome maintenance (lysosome, phagosome proteins, carbonic anhydrases, and Vtype ATPases) and reactive oxygen species (ROS) detoxification enzymes were assessed due to their known role in coral bleaching (Table S5 and See Data S1). Some proteins may be part of multiple pathways but are presented only once in the results. Figure 3B summarizes the results presented below.

### 2.6 Shared Proteome Responses in Coral Hosts

#### 2.6.1 Carbohydrate Metabolism

All three coral species exhibited downregulation of carbohydrate metabolism under thermal stress, with 39 proteins differentially abundant. However, only two proteins were shared across species, although they were differentially abundant at different timepoints. Malonate‐semialdehyde dehydrogenase (ALDH6A1, K00140), involved in propionate metabolism, showed decreased abundance at TP1 in both *A. hyacinthus* (FC = −0.52, *p*‐value = 0.0086) and *P. lobata* (FC = −0.72, *p*‐value = 0.011), and at TP2 in *P. lobata* (FC = −0.77, *p*‐value = 0.025). Aconitate hydratase (ACO, K01681) exhibited a divergent pattern, with increased abundance in *S. pistillata* at TP1 (FC = 1.09, *p*‐value = 0.032) and decreased abundance in *P. lobata* at TP2 (FC = −0.58, *p*‐value = 0.044). The remaining 37 proteins were species‐specific (Figure 3B).

#### 2.6.2 Energy Metabolism

Downregulation of energy metabolism was observed in all three species. Key proteins in oxidative phosphorylation pathways, including ATPases, NADHs, and cytochrome proteins, were differentially abundant. In *A. hyacinthus* at TP1, ATPeF1A (K02132, FC = −0.50, *p*‐value = 0.037) and ATPeF1B (K02133, FC = −0.53, *p*‐value = 0.035) both showed decreased abundance. Additionally, the V‐type ATPase (VHA) genes ATPeV1G (K02152, FC = −1.57, *p*‐value = 0.027) and ATPeV1F (K02151, FC = −1.44, *p*‐value = 0.031) also showed decreased abundance at TP1. None of these proteins were statistically significant at TP2. Different proteins related to energy metabolism were differentially abundant in *P. lobata* . At TP1, NADH dehydrogenase subunit NDUFA5 (K03949, FC = −0.60, *p*‐value = 0.035) showed decreased abundance. A stronger trend was observed in the same pathway at TP2, wherein ATPeFG (K02140, FC = −0.79, *p*‐value = 0.042), NDUFS4 (K03937, FC = −0.67, *p*‐value = 0.010), and ATPeV1F (FC = −1.49, *p*‐value = 0.041) all showed decreased abundance in *P. lobata* . No significant shifts in oxidative phosphorylation proteins were observed in *S. pistillata* .

#### 2.6.3 Amino Acid Metabolism

Amino acids play an essential role in the coral‐algal symbiosis and are catabolized by the host under thermal stress. Glutamine synthetase (GLNA; K01915) showed decreased abundance in *A. hyacinthus* at both TP1 (FC = −0.98, *p*‐value = 0.028) and TP2 (FC = −1.09, *p*‐value = 0.020). In *S. pistillata* , two copies of GLNA were detected, both of which showed decreased abundance at TP1 (FC = −0.64, *p*‐value = 0.0043 and FC = −0.74, *p*‐value = 0.00032). No shifts in GLNA abundance were observed in *P. lobata* at TP1; however, a significant decrease was observed at TP2 (FC = −2.35, *p*‐value = 0.0051). We also assessed the abundance of glutamate dehydrogenase (GDH). One GDH protein (K00262) was detected in *S. pistillata*, which showed a significant decrease at TP1 (FC = −0.50, *p*‐value = 0.0029). In *A. hyacinthus* , a different GDH protein (K00261) was identified that showed increased abundance at TP2 (FC = 0.80, *p*‐value = 0.016). In *P. lobata* , no significant shifts in GDH abundance were detected. Lastly, we assessed 4‐hydroxyphenylpyruvate dioxygenase (HPPD), which showed a significant increase only in *P. lobata* at TP2 (K00457, FC = 0.68, *p*‐value = 0.028).

#### 2.6.4 Protein Folding, Sorting, and Degradation

Elevation of heat shock protein (HSP) abundance is one of most conserved responses in corals under thermal stress (Molinari et al. 2025) and showed significant responses in all three species. At TP1, three HSPs showed a significant increase in *S. pistillata* only: HSPA5 (K09490, FC = 1.16, *p*‐value = 0.0013), HSP90B (K09487, FC = 0.96, *p*‐value = 0.00057), and HSP90A (K04079, FC = 0.78, *p*‐value = 0.007). At TP2, significant increases were observed in both *A. hyacinthus* and *P. lobata* for HSPA5 (FC = 0.60, *p*‐value = 0.016; FC = 0.57, *p*‐value = 0.028, respectively) and HSP90B (FC = 1.01, *p*‐value = 0.00086; FC = 1.26, *p*‐value = 0.0022). Additionally, DNAJ proteins (HSP40 family) showed similar patterns: at TP1, two proteins increased in *S. pistillata* , DNAJC3 (K09523, FC = 1.22, *p*‐value = 0.00083) and DNAJA2 (K09503, FC = 2.05, *p*‐value = 0.010). At TP2, *A. hyacinthus* also showed increased abundance of DNAJC3 (FC = 1.07, *p*‐value = 0.016). Another chaperone protein, Calreticulin (CALR, K08057), showed a staggered yet consistent response. At TP1, it showed increased abundance in *P. lobata* (FC = 0.71, *p*‐value = 0.026) and *S. pistillata* (FC = 0.89, *p*‐value = 0.0065). At TP2, we observed a significant increase in both *A. hyacinthus* (FC = 0.70, *p*‐value = 0.0058) and *P. lobata* (FC = 1.02, *p*‐value = 0.0081). We also observed significant shifts in proteasome subunit proteins involved in degradation (See Data S1). Together, these results indicate that folding, sorting, and degradation pathways were consistently increased during thermal stress in the three species but occurred at different stages in each. Furthermore, numerous proteins in this pathway have been extensively associated with coral thermal stress responses (Kenkel and Matz 2016; Nakamura et al. 2011; Rosic et al. 2011).

#### 2.6.5 Lipid Metabolism

Shifts in lipid content are known markers of coral thermal stress. We observed two key fatty acid biosynthesis genes that showed conserved and significant responses to thermal stress: long‐chain‐fatty‐acid–CoA ligase (ACSBG, K15013) and long‐chain acyl‐CoA synthetase (ACSL, K01897). At TP1, decreased abundance was observed in two ACSBG proteins in *A. hyacinthus* (FC = −0.60, *p*‐value = 0.014; FC = −0.86, *p*‐value = 0.043). At TP1, *P. lobata* also showed decreased abundance of ACSL (FC = −1.18, *p*‐value = 0.019). At TP2, we observed a similar pattern in *A. hyacinthus* , with two ACSBG proteins showing decreased abundance (FC = −0.61, *p*‐value = 0.033; FC = −1.02, *p*‐value = 0.046), and ACSL also decreasing (FC = −0.93, *p*‐value = 0.029). In contrast, at TP2, *P. lobata* showed increased abundance of ACSL (FC = 0.67, *p*‐value = 0.000048).

#### 2.6.6 Symbiosome Maintenance Proteins

Overall, symbiosome maintenance pathways showed species and timepoint‐specific disruption under thermal stress, with early activation of lysosomal degradation in *A. hyacinthus* and *S. pistillata* and more variable or delayed responses in *P. lobata* , consistent with differential regulation of host–symbiont stability during stress. We observed shifts in several key proteins potentially involved in symbiosomal maintenance. Two cathepsin proteins: CTSL (K01365) and CTSB (K01363), associated with the lysosome, were differentially abundant. At TP1, CTSB showed increased abundance in *A. hyacinthus* (FC = 0.79, *p*‐value = 0.043) and *S. pistillata* (FC = 1.02, *p*‐value = 0.015). CTSL also showed increased abundance in *A. hyacinthus* (FC = 1.59, *p*‐value = 0.046) and *S. pistillata* (FC = 0.58, *p*‐value = 0.025). No significant response was observed in *P. lobata* at TP1. At TP2, CTSB showed increased abundance in both *A. hyacinthus* (FC = 0.93, *p*‐value = 0.028) and *P. lobata* (FC = 1.51, *p*‐value = 0.028). CTSL showed a mixed response in *P. lobata* , with one copy increasing (FC = 1.50, *p*‐value = 0.0015) and another decreasing (FC = −1.97, *p*‐value = 0.022).

Niemann‐Pick C2 (NPC2, K13443), a lysosome‐associated protein, showed decreased abundance in *A. hyacinthus* at TP2 (FC = −2.04, *p*‐value = 0.0069). Neutrophil cytosolic factor 2 (NCF2), involved in phagosomes and ROS production, showed decreased abundance at TP2 in both *A. hyacinthus* (FC = −0.88, *p*‐value = 0.045) and *P. lobata* (FC = −1.63, *p*‐value = 0.012). Carbonic anhydrase 2 (CA2, K18245) showed decreased abundance in *A. hyacinthus* at TP1 (FC = −2.26, *p*‐value = 0.025) and TP2 (FC = −3.61, *p*‐value = 0.0018). Additionally, suppression of the transcription factor NF‐κB (K02580) has been implicated to play a role in establishing and maintaining symbiosis (Mansfield et al. 2017), however, in this study we observed an increased abundance in *A. hyacinthus* at both TP1 (FC = 0.63, *p*‐value = 0.025) and TP2 (FC = 1.43, *p*‐value = 0.025), suggesting a potential role in endosymbiont dysbiosis. No significant shifts were observed in the other species (for additional signaling proteins see Data S1).

### 2.7 Differences in Metabolomic Biomarkers Across Species

**FIGURE 4:**
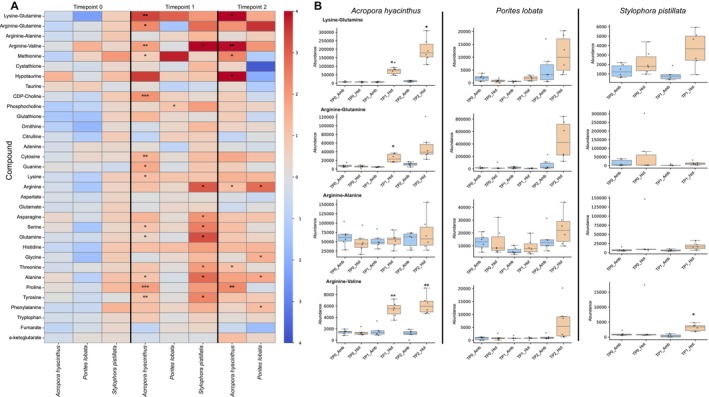
Differential abundance of polar metabolites. (A) Heatmap of targeted metabolite responses across coral species and time points, with colors indicating relative changes in abundance; asterisks denote adjusted significance levels (* adj‐*p*‐value < 0.05; ** adj‐*p*‐value < 0.01; *** adj‐*p*‐value < 0.001). (B) Boxplots showing abundance patterns of the four dipeptides across time points and coral species under ambient and thermal stress conditions.

We investigated metabolomic data to validate previous findings regarding dipeptides, amino acids, and antioxidants. The dipeptides KQ, RQ, RA, and RV accumulate under thermal stress (Williams, Chiles, et al. 2021). In *A. hyacinthus* at TP1, KQ (FC = 3.40, adj‐*p*‐value = 0.0049), RQ (FC = 2.30, adj‐*p‐*value = 0.026), and RV (FC = 1.86, adj‐*p*‐value = 0.0049) all significantly increased in abundance. At TP2, the same dipeptides remained elevated. In *S. pistillata* at TP1, only RV showed a significant increase (FC = 6.73, adj‐*p*‐value = 0.029), whereas the other dipeptides also had increased abundances; these were not statistically significant. In *P. lobata* , no dipeptides were significantly differentially abundant at either TP1 or TP2, though increased fold‐changes were observed at TP2 (Figure 4B and Table S6). Consistent with the proteomic patterns, dipeptide responses were temporally staggered across species and broadly mirrored known thermal tolerance differences, with earlier accumulation in *A. hyacinthus* and *S. pistillata* and comparatively muted responses in *P. lobata* (non‐significant increase).

We assessed the abundance of well‐characterized antioxidant metabolites (Williams, Pathmanathan, et al. 2021). Each coral holobiont showed a distinct profile of stress metabolite accumulation (Table S6b). In *A. hyacinthus* , methionine had an increased abundance at both TP1 (FC = 0.74, adj‐*p‐*value = 0.046) and TP2 (FC = 2.34, adj‐*p*‐value = 0.018). Hypotaurine also had an increased abundance at TP2 in *A. hyacinthus* (FC = 11.23, adj*‐p*‐value = 0.035). CDP‐choline also had an increased abundance in *A. hyacinthus* at TP1 (FC = 2.10, adj‐*p*‐value = 0.00030). However, these shifts were exclusive to *A. hyacinthus* only. Whereas phosphocholine was the only antioxidant metabolite that had an increased abundance at TP1 in *S. pistillata* (FC = 1.93, adj*‐p*‐value = 0.054) and a significant increase in *P. lobata* (FC = 1.03, adj*‐p*‐value = 0.027). A temporal and species‐specific response was also noted in the antioxidant metabolites, suggesting diverged mechanisms to thermal stress responses with respect to metabolite abundance.

In addition, all three coral species showed increased abundance of amino acids (Figure 4A and Table S6c). In *A. hyacinthus* , nine amino acids increased at TP1 (seven were statistically significant, adj‐*p*‐value < 0.05), and nine remained increased at TP2 (four were significant). In *S. pistillata* , 12 amino acids had an increased abundance (seven were significant). In *P. lobata* , seven amino acids had an increased abundance (four were significant). Of note only two amino acids (arginine and alanine) had an increased abundance in all three species, though this was temporally varied. Arginine had an increased abundance at TP1 in *S. pistillata* (FC = 3.01, adj‐*p‐*value = 0.035) and subsequently at TP2 in *A. hyacinthus* (FC = 1.12, adj‐*p‐*value = 0.036) and *P. lobata* (FC = 2.78, adj‐*p‐*value = 0.028). Alanine had an increased abundance at TP1 in *A. hyacinthus* (FC = 1.00, adj‐*p‐*value = 0.027) and *S. pistillata* (FC = 2.81, adj‐*p‐*value = 0.035), and at TP2 in *P. lobata* (FC = 1.69, adj‐*p‐*value = 0.017).

## 3 Discussion

### 3.1 Overall Trends in Coral Physiology

The three sympatric coral species investigated in this study ( *Acropora hyacinthus* , *Porites lobata* , and *Stylophora pistillata* ) have well‐documented differences in thermal stress response. *A. hyacinthus* is thermally sensitive, *P. lobata* is thermally resilient, and *S. pistillata* displays a low to moderate thermal stress tolerance (Burn et al. 2023; Fitt et al. 2009; Manalili et al. 2025; Meziere et al. 2022; Pratchett et al. 2020). In the experimental tanks, temperatures were ramped from the local (GBR) maximum monthly mean (28.38°C) to 32.4°C over 5 days, followed by 4 days at peak temperature. All three corals exhibited expected physiological responses to heat stress, including reduction in endosymbiont abundance (Glynn 1991; Jokiel and Coles 1977) and photosynthetic efficiency (*F*_*v*_ */F*_*m*_) (Warner et al. 1999, 1996).

Consistent with the expected resilience levels, *A. hyacinthus* bleached first, indicated by reduced endosymbiont abundance upon initial (TP1, fold change = −1.26) stress and subsequently, a more significant loss under prolonged (TP2, fold change = −1.88) stress. A 19.16% decrease in photosynthetic efficiency was also observed at TP2. This rapid, more severe bleaching response of *A. hyacinthus* is consistent with the known temperature sensitivity of the *Acropora* genus, including rapid photo‐physiological impairment of symbionts and early activation of host cellular stress pathways under acute thermal stress (Loya et al. 2001; Manalili et al. 2025; Sakai et al. 2019; Suggett et al. 2017). In contrast, *P. lobata* had only a slight decline (non‐significant) in endosymbiont abundance at initial stress (TP1 = −0.01) and a more profound decline at prolonged stress (TP2 = −2.25, though not statistically significant). A 7.65% decrease in photosynthetic efficiency was observed at TP2. Lastly, in *S. pistillata* colonies exhibited polyp bail‐out, a rapid tissue loss response that is a severe stress reaction in this species (Chuang et al. 2021; Schweinsberg et al. 2021). Reduced endosymbiont cell abundance was noted at initial stress (−1.3 fold‐change) and significant reduction in photosynthetic efficiency was also observed (20.70%).

The ITS2 profiles of the algal symbionts differed across coral species, potentially influencing the thermal sensitivity of their hosts. However, all symbionts were in the genus *Cladocopium*, which is considerably less heat‐tolerant than *Durusdinium* (Berkelmans and van Oppen 2006). Reported differences among *Cladocopium* subtypes rely mainly on *F*_*v*_ */F*_*m*_ data (Fisher et al. 2012; Ziegler et al. 2015) and lack mechanistic and host‐independent insights. Furthermore, a minor *Gerakladium* signal (< 1% abundance) was detected in one sample; however, such low‐level representation is unlikely to reflect a stable or functionally significant symbiont within the holobiont. Lastly, the three corals were collected from the same reef in the GBR and likely had similar historical environmental stress profiles, helping to minimize variability due to differences in past stress exposure (Barshis et al. 2013; Kenkel and Matz 2016; Morgan et al. 2017; Oliver and Palumbi 2011; Risk and Edinger 2011; Rogers et al. 2016; Safaie et al. 2018).

We assessed both the endosymbiont and host proteomes and observed tank effects in each. In the coral hosts, these effects were minimal, with only a few proteins showing differential abundance at TP0 (File S1). In contrast, the endosymbiont proteome exhibited more pronounced variability. Endosymbiont protein abundances were normalized to account for bleaching‐induced endosymbiont cell loss, however, this adjustment revealed pronounced tank effects. Specifically, we observed a decline in protein abundance in ambient samples over time (File S2), despite the absence of thermal stress. This pattern likely contributed to the observation of inflated responses in the endosymbiont proteome. Coral fragments were collected from the reef and acclimated in outdoor flow‐through tanks for approximately 3 weeks before being transferred to indoor experimental tanks, where they were acclimated for one additional week prior to heat stress exposure. Tank effects were not associated with any single tank or treatment and appeared to be randomly distributed across experimental units, suggesting they were not driven by identifiable tank‐specific factors such as flow or temperature. We therefore attribute these effects primarily to the relatively short acclimation period to the indoor environment, particularly the shift in light intensity (outdoor to indoor), which may have disproportionately impacted the photosynthetic endosymbionts. As a result, we excluded the endosymbiont proteome from further analysis and focused instead on host‐specific responses to heat stress. Physiological indicators, including reductions in endosymbiont abundance and photosynthetic efficiency, supported the occurrence of bleaching. Together, these findings suggest a decline in photosynthate transfer from endosymbionts to the host under thermal stress, putatively shifting the endosymbiont to a more parasitic state, that is, hoarding photosynthates, a response observed in previous coral bleaching studies (Baker et al. 2018; Davy et al. 2012; Grottoli et al. 2006; Rädecker et al. 2021).

### 3.2 Pathway and Protein Level Responses Are Consistent With Diverged Ecological Outcomes to Thermal Stress

**FIGURE 5:**
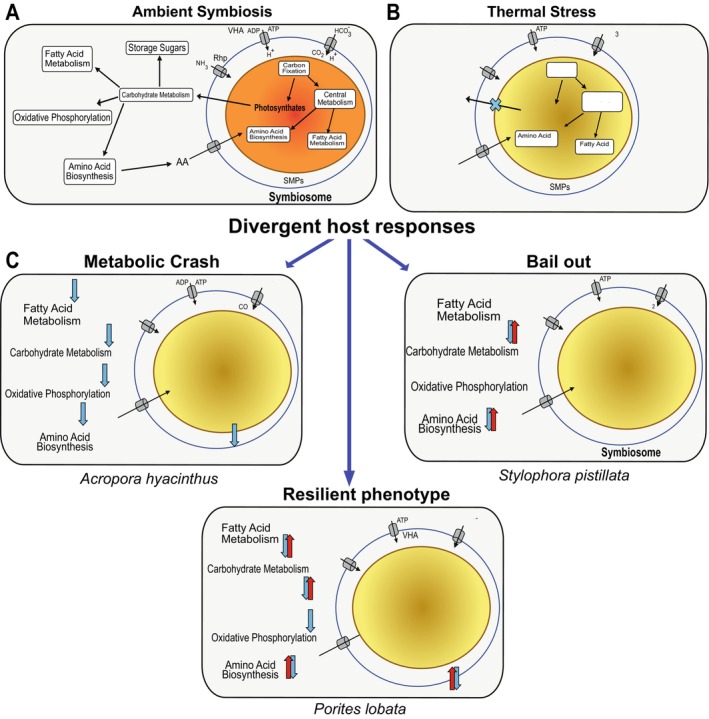
Divergent outcomes of thermal stress in three GBR coral species. Conceptual overview of host‐symbiont metabolic interactions and host responses to thermal stress. (A) Schematic of a stable coral‐endosymbiont symbiosis characterized by reciprocal nutrient exchange and transfer of photosynthates to the host. (B) Disruption of symbiont function under thermal stress, resulting in reduced photosynthate transfer to the host. (C) Divergent metabolic responses among the three coral species, that is, *A. hyacinthus* (metabolic crash), *S. pistillata* (bail‐out), and *P. lobata* (resilient phenotype).

Corals depend on their algal symbionts to meet metabolic needs, thus, reduction in symbiont‐derived photosynthate can severely impact holobiont fitness (Gordon and Leggat 2010; Tremblay et al. 2012). Our data reveal that different coral species exhibit distinct proteomic responses to thermal stress and the associated decline in photosynthate transfer (Figure 5). However, the timing of these protein shifts was different between the species, likely due to differences in bleaching sensitivity, that is, more susceptible versus more resilient. Differences in bleaching sensitivity likely influence the timing of proteomic responses, with more sensitive species undergoing earlier and broader proteomic differentiation, whereas less sensitive species exhibit delayed or more regulated responses. *A. hyacinthus* demonstrated early and extensive proteomic shifts, consistent with its high thermal sensitivity. Upon initial thermal stress, this species showed dramatic decreases in protein abundance across a substantial portion of its proteome (34.6% of proteins based on fold‐change; Figure S3), indicating a strong early proteomic response to thermal stress. Upon prolonged thermal stress, additional shifts were observed, with 75 proteins increasing and 101 decreasing in abundance, a pattern that is consistent with proteomic responses reported previously for *Acropora* under elevated temperature (Petrou et al. 2021; Ricaurte et al. 2025).

*P. lobata* displayed a more gradual and delayed response, wherein under initial stress a moderate response was observed. However, with prolonged stress, the response became more prominent, resembling the pattern seen in *A. hyacinthus*, but occurring later. Notably, many proteins in *P. lobata* showed mixed responses across different copies, suggesting complex pathway regulation. *S. pistillata* exhibited a unique response pattern, showing both increases and decreases in protein abundances reflecting a mixed but active stress‐response strategy, before ultimately undergoing polyp bail‐out, approximately 24 h after sampling.

Consistent with their known ecological differences in thermal tolerance, we observed divergent responses at the pathway level among the three coral species. We evaluated the differential abundance of proteins across 25 KEGG pathways, 21 of which exhibited species‐specific patterns. Among these, the “cell growth and death” pathway, which includes apoptosis and has been previously linked to coral thermal stress responses (Ainsworth et al. 2011), showed the most pronounced divergence and thus serves as a prime example (Figure 3A). In *A. hyacinthus* , this pathway was downregulated during initial stress but upregulated under prolonged stress. In contrast, *P. lobata* exhibited a mixed initial response that shifted to downregulation under longer exposure to stress. *S. pistillata* also showed a mixed response at initial stress. These contrasting trajectories highlight the distinct stress‐response strategies in these species and align with their known phenotypes. Such divergent responses underscore the challenges in identifying universal biomarkers of coral stress (see below).

Consistent with the known thermal sensitivity of species in the genus *Acropora* (Sakai et al. 2019; Singh et al. 2019), we observed that in *A. hyacinthus* , 16 pathways were downregulated under initial thermal stress, indicative of a broad metabolic collapse (Figure 5). The host appeared to enter an energy‐depleted state earlier than the other two coral species, putatively suggesting a higher reliance on endosymbiont derived photosynthates. We hypothesize that in response to reduced photosynthate transfer, the host shifts energy away from endosymbiont maintenance, reflected by decreased abundance of carbon supply proteins (e.g., CA2) (Bertucci et al. 2013; Zoccola et al. 2015) and increased abundance of proteins associated with symbiosis. For example, NF‐κB which is downregulated during the formation of symbiosis, had an increased abundance under thermal stress (Mansfield et al. 2017). These trends were reflected at the physiological level, wherein *A. hyacinthus* was the first of the three species to exhibit substantial endosymbiont loss. Under prolonged stress, the proteomic profile shifted from widespread downregulation toward a mixed pattern of protein increases and decreases, reflecting proteomic reorganization under sustained stress rather than physiological recovery, and consistent with increased reliance on endogenous carbohydrate, lipid, and amino acid reserves to maintain metabolic function following dysbiosis (Petrou et al. 2021; Rodrigues and Grottoli 2007).

In *P. lobata* , a species known for its relative resilience to thermal stress (Huang et al. 2025; Rivera et al. 2022), relatively few pathways were affected during initial stress. This finding is consistent with physiology, whereby no significant loss of endosymbiont cells was observed, and reduced photosynthetic efficiency was noted only under prolonged stress. Furthermore, under prolonged stress at an individual protein level, *P. lobata* had numerous instances of mixed responses (i.e., one copy of a protein had increased abundance, and another showed a decrease). For example, at TP2 the CTSL protein in *P. lobata* had one gene copy that increased in encoded protein abundance and another that decreased (Figure 3B). These mixed gene‐copy translation responses may indicate a compensatory mechanism in *P. lobata* which may be responsible for the higher thermal tolerance in this genus. Species in the genus *Porites* have larger nuclear genomes and subsequently encode more genes (30,000–60,000 genes) (Stephens et al. 2022), whereas other genera and species have smaller genomes and gene inventories, such as *Acropora* (~20,000 genes) (Shinzato et al. 2021) and *Stylophora pistillata* (24,833 genes) (Voolstra et al. 2017). We speculate that the larger genomic repertoire and higher gene‐copy number in *Porites* species may enable a more complex and flexible stress response, potentially underpinning their thermal resilience phenotype. Furthermore, lineage‐specific gene family expansions have been documented in *Porites*, including duplications of stress‐responsive genes, which are transcriptionally responsive to elevated temperature and may contribute to the high stress tolerance observed in this genus (Shinzato et al. 2021).

Physiologically, *S. pistillata* exhibited a bail‐out response under thermal stress (Schweinsberg et al. 2021). Unsurprisingly, at the molecular level we observed distinctive responses compared to the other two species. We observed many pathways upregulated at initial stress (7 of the 25), which included transcription and translation and were species‐specific responses in *S. pistillata* . A study that performed salinity induced bail‐out in the coral *Pocillopora acuta* found key enriched responses in signaling pathways (Chuang et al. 2021; Chuang and Mitarai 2020). Proteins in signaling pathways were relatively stable; however, this may be due to the measurements being made prior to bail‐out and not during active bail‐out or may simply reflect the smaller number of proteins obtained from *S. pistillata* when compared to the other two coral species. Shared with *P. acuta* , we found an increased abundance in proteasome associated proteins that may be involved in proteolysis (PSMA3, PSMA4, and PSMD1) (Chuang and Mitarai 2020) (see Data S1).

### 3.3 Diverged Protein Responses Even in Pathways With a Conserved Response

Only four of the 25 pathways exhibited a conserved response across all three coral species (Figure 3A). Within these, two key trends emerged. First, although the direction of change was consistent across species (e.g., reduced abundance), the timing varied. For example, the energy metabolism pathway was downregulated in *A. hyacinthus* and *S. pistillata* during initial stress, but not until prolonged stress in *P. lobata* (Figure 3A). These conserved, yet temporally staggered, responses align with known bleaching susceptibility, and subsequently endosymbiont photosynthate limitation. These temporal offsets likely reflect species‐specific differences in the intensity of stress responses, with more bleaching‐susceptible species mounting earlier and more pronounced pathway‐level changes (e.g., *A. hyacinthus* and *S. pistillata* ), whereas more tolerant species exhibit delayed and more regulated responses (e.g., *P. lobata*). Declines in carbohydrate and energy metabolism have been widely documented, although the magnitude and timing differ across species and experimental conditions (Haydon et al. 2023; Petrou et al. 2021). Lipid metabolism, a commonly used biomarker of coral bleaching, was also consistently downregulated, consistent with prior studies (Ermolenko and Sikorskaya 2021; Grottoli et al. 2004; Imbs and Dembitsky 2023). In contrast, protein folding, sorting, and degradation pathways were upregulated, which consist of heat‐shock proteins, potentially reflecting increased demand for the turnover and clearance of misfolded or damaged proteins under stress (Williams, Pathmanathan, et al. 2021).

The second trend we observed was that even within pathways exhibiting a conserved overall response, though temporally varied, the specific proteins driving that response differed across species. For instance, carbohydrate metabolism was consistently downregulated across species; only two (ALDH6A1 and ACO) of the 39 differentially abundant proteins were shared between any two species. ALDH6A1 was differentially abundant in both *A. hyacinthus* and *P. lobata* , whereas ACO was affected in *S. pistillata* and *P. lobata* but showed divergent expression patterns. The remaining 37 proteins were differentially abundant in only one species (Figure 3B). These patterns indicate that, during bleaching, corals undergo species‐specific metabolic rewiring, relying on different protein‐level adjustments to converge on similar pathway‐level responses.

### 3.4 Shared Protein and Metabolite Responses Under Thermal Stress

#### 3.4.1 Conserved Protein Responses

In this section, we examined the individual proteins that exhibited a conserved response in the three coral species. We first focused on canonical stress response proteins, commonly observed in corals and other metazoans. Heat shock proteins (HSPs) are well‐established markers of thermal stress (Rosic et al. 2011; Sharp et al. 1997). These proteins are widely used as biomarkers of stressand play a central role in promoting survival under elevated temperatures in corals (Cleves et al. 2020; Louis et al. 2017). In our study, HSPA5 (also known as HSP70) and HSP90B showed conserved upregulation across all species. Notably, this response was temporally staggered, wherein *S. pistillata* exhibited increased abundance at initial stress, whereas *A. hyacinthus* and *P. lobata* showed increases later in the stress period. This temporally staggered abundance likely reflects species‐specific thermal thresholds and activation dynamics of stress‐signaling pathways. These patterns appeared to be consistent with other studies performed on these coral genera (Maor‐Landaw and Levy 2016; Seveso et al. 2020). We also observed a conserved increase in calreticulin (CALR), a calcium‐binding endoplasmic reticulum chaperone involved in protein folding and quality control (Fucikova et al. 2021). CALR complements HSP activity by contributing to endoplasmic reticulum protein folding and chaperone‐mediated quality control during thermal stress, consistent with coral studies showing coordinated activation of ER chaperones and heat shock proteins under elevated temperatures (Ishibashi et al. 2023; Ruiz‐Jones and Palumbi 2017).

We also noted conserved proteins that are not directly involved in thermal stress responses but have been implicated in previous studies. One such protein is glutamine synthetase (GLNA), which is involved in ammonium assimilation. GLNA abundance decreased in all three coral species; GLNA has been previously observed to have a decreased abundance at the transcriptomic level in *S. pistillata* from the Red Sea (Rädecker et al. 2021), suggesting a conserved response across species and geographic locations. Ammonium assimilation plays a central role in the coral‐dinoflagellate symbiosis, with the host supplying ammonia to the endosymbiont (Pernice et al. 2012). We propose that reduced GLNA abundance may indicate diminished host assimilation of ammonium, which could allow greater availability or transfer of inorganic nitrogen to the endosymbiont during stress, consistent with altered nitrogen partitioning observed during bleaching (Rädecker et al. 2021).

Lastly, we observed a conserved response for two lysosomal cysteine proteases, cathepsin proteins CTSB and CTSL. Cathepsins, including CTSB and CTSL, have been linked to general cellular stress pathways, such as apoptosis and oxidative stress, which are well‐established components of coral bleaching responses (Louis et al. 2017; Schwarz et al. 2013). Increased expression of CTSB under copper and thermal stress has been observed in corals and is suggested to play a role in the ROS response (Louis et al. 2017; Schwarz et al. 2013). Increased CTSL expression has been previously associated with pathogenic infections and thermal stress in corals (Louis et al. 2017; Skutnik et al. 2020; Wright et al. 2015).

#### 3.4.2 Conserved Metabolomic Responses

In this study, we used polar metabolomics to identify and validate previously proposed metabolite biomarkers of thermal stress. Consistent with the physiological and proteomic responses, a notable temporal variation was observed in the metabolomic data. Within the coral holobiont, the host and endosymbiont share the same amino acid pool. The coral host supplies ammonia to the endosymbiont where it is assimilated and amino acids are transferred to the coral host (Gordon and Leggat 2010; Martinez, Grover, Baker, and Ferrier‐Pagès 2022). Under thermal stress, increases in free amino acids are thought to serve two primary functions within the coral holobiont. First, amino acid availability can help sustain the host‐symbiont relationship because corals that actively transfer amino acids to their endosymbionts exhibit slower bleaching dynamics (Martinez, Grover, and Ferrier‐Pagès 2022; Martinez, Grover, Baker, and Ferrier‐Pagès 2022; Martinez et al. 2020). Second, following symbiont dysfunction or loss, amino acid catabolism may support host energy demands by partially compensating for reduced photosynthate supply (Rädecker et al. 2021). Furthermore, increased amino acid catabolism for host sustenance produces excess ammonia which seeps into the symbiosome and decouples nutrient exchange by shifting symbionts from mutualistic to self‐serving metabolism (Rädecker et al. 2021).

We observed increased free amino acid abundances in all three coral species, although the timing of these responses was species‐specific. Whereas elevated amino acid pools can be associated with enhanced heterotrophic feeding (Houlbrèque and Ferrier‐Pagès 2009; Petrou et al. 2018), no heterotrophic supplementation (e.g., *Artemia*) was provided during the experiment, suggesting that these changes reflect endogenous metabolic restructuring rather than external nutrient input. Furthermore, the increase in free amino acids also appeared to be temporally staggered and consistent with the known thermal sensitivities of the coral species. Two amino acids, alanine and arginine, appeared to have a conserved increased abundance in all three coral species, making them interesting candidates for future biomarker studies.

In *A. hyacinthus* , increased amino acids coincided with early endosymbiont loss, suggesting they may supplement host metabolism under stress. Likewise, in *S. pistillata* , the increased amino acid pool may be used to serve as a source of nutrition post bail‐out (Houlbrèque and Ferrier‐Pagès 2009; Petrou et al. 2018). In contrast, *P. lobata* maintained stable endosymbiont abundances even under prolonged stress, implying that the elevated amino acid pool may be transferred to the symbiont to sustain the symbiosis as has been noted in other species (Martinez, Grover, Baker, and Ferrier‐Pagès 2022; Martinez et al. 2020).

Dipeptides are markers of stress in other model systems (Williams, Pathmanathan, et al. 2021) and show great promise in corals. Little is known however about the pathways involved in their synthesis and downstream functions. Stable isotope analysis demonstrates large differences in the turnover rates of dipeptides and free amino acids, suggesting that these have distinct biosynthetic mechanisms in corals (Chiles et al. 2022). We observed a conserved increase in dipeptide abundance across species, with all four dipeptides exhibiting positive fold changes under thermal stress. Although only KQ, RQ, and RV were significantly increased in *A. hyacinthus* , and RV was significant in both *S. pistillata* and *P. lobata* . The consistent increase in dipeptide abundance across studies and coral species highlights dipeptides as promising biomarkers of thermal stress (Chiles et al. 2022; Williams, Chiles, et al. 2021). Like the patterns observed in the proteome, dipeptide responses were temporally staggered, reflecting species‐specific dynamics.

### 3.5 Study Limitations

Despite careful experimental design, tank effects were observed in the coral and algal endosymbiont proteomes, which is a persistent issue in coral research (McLachlan et al. 2022; Molinari et al. 2025). These effects were not driven by a single tank condition but were consistent in magnitude across replicate tanks. We believe that these effects were primarily driven by the short acclimation period utilized (~1 week) prior to thermal stress treatment. After normalization of the endosymbiont proteome using changes in cell abundance, the magnitude of this effect became even more apparent (File S1), which disallowed use of the endosymbiont data. Notably, colonies maintained under ambient, non‐stress conditions still exhibited substantial shifts in endosymbiont proteomes across all three coral species, as shown by changes in median protein abundance. We interpret these shifts as evidence of stress induced by ongoing acclimation to indoor tanks, that is, different light intensities compared to outdoor tanks, consistent with previous observations that Symbiodiniaceae are highly sensitive to subtle environmental fluctuations (Castro‐Sanguino et al. 2024; Mass et al. 2010). In contrast, tank effects on the coral host proteome were minimal. Whereas it is plausible that alterations in the endosymbiont proteome would influence the coral host principal component analysis, comparisons of median protein abundance and overall proteome composition indicated limited host‐level variation among tanks (File S2). In addition, we obtained different coverage levels in the proteome, wherein significantly fewer proteins were obtained in *S. pistillata* relative to the other species. Because sample processing, instrument settings, and analytical workflows were consistent across species, the basis for this reduced coverage remains unclear and may reflect a combination of species‐specific proteomic characteristics and analytical limitations rather than a single identifiable factor. Finally, limited biological replication constrained statistical power vis‐à‐vis individual proteins, underscoring the need for increased replication in future coral proteomic studies. At a fundamental level, we emphasize the value of implementing pilot studies to ascertain appropriate acclimation periods for all genotypes and increasing replicates to achieve data consistency when using multi‐omics methods.

## 4 Conclusion

In this study we subjected three sympatric coral species *A. hyacinthus* (sensitive), *P. lobata* (resilient), and *S. pistillata* (bail‐out) with known ecological divergent responses to thermal stress. Thermal stress was associated with declines in endosymbiont cell density and reduced photosystem II efficiency (*F*_*v*_/*F*_m_), consistent with canonical bleaching‐associated physiological patterns. However, we observe a clear diverged response in the proteome with only four pathways having a consistent response in all three coral species. These four pathways have been demonstrated to be involved in coral stress responses, for example energy metabolism, lipid metabolism, and protein folding, sorting, and degradation. However, across species, we observed temporally staggered responses that reflect known differences in thermal tolerance among these taxa. Even within the four pathways, we note that only a handful of proteins had a consistent response across species. From the metabolomic data we note that dipeptides consistently show an increased accumulation in all three species upon exposure to thermal stress; however, some species showed a stronger response than others, further supporting the species‐specific nature of these responses. These species‐specific and temporally staggered responses were reflected in coral thermal tolerance capabilities. Overall, in *A. hyacinthus* we observe a sharp initial response to thermal stress followed by proteomic reorganization at prolonged stress, whereas in *P. lobata* we observe a mixed response during prolonged stress, and lastly, in *S. pistillata* we observed an initial stress response which was followed by host bail‐out.

## 5 Methods

### 5.1 Test Species

A single large colony of *Acropora hyacinthus* (neat morphotype), *Stylophora pistillata* , and *Porites lobata* was sampled from Davies Reef (−18°49′30″ S, 147°38′42″ E) on the central GBR in February 2023 (Permit Number: G19:43148) (Figure 1). Only one genotype was used to avoid the possibility of stochastic inter‐colony variation in the stress proteome and metabolome (Chille et al. 2024), however, this design necessarily limits the generalizability of species‐level inferences. These species were selected to represent different morphologies, metabolic rates, and heat stress sensitivities: *Acropora hyacinthus*‐sensitive, tabulate, branching; *Stylophora pistillata*‐low/moderate tolerance, branching; *Porites lobata*‐tolerant, massive (Loya et al. 2001; van Woesik et al. 2011).

### 5.2 Experimental Design

Each colony was subdivided into 36 fragments (~7 cm^2^), mounted on experimental racks, and allowed to recover for 3 weeks prior to the start of the experiment at the National Sea Simulator (SeaSim) at the Australian Institute of Marine Science (AIMS) in outdoor flow through tanks replicating Davies Reef conditions (28.0°C, ca. ~250 μmol photons m^−2^ s^−1^). Prior to heat stress treatments, fragments were randomly assigned across six 50 L experimental flow through tanks (*n* = 18 per tank, 6 fragments per species), three for each of the two experimental treatments and acclimated for 7 days before initiating heat stress. Fragments were exposed to two treatments: a temperature treatment of 32.4°C and a control maintained at 28.38°C (Maximum Monthly Mean at Davies Reef). The MMM is defined as the maximum sea surface temperature (SST) of the 12 monthly mean values for the years 1985–1990 and 1993 (Heron et al. 2014) temperature ramping increased from 28.38°C to 32.4°C, over 5 days (0.8°C/day) for treatment tanks. The heat stress experiment ran for a further 4 days concluding after 9 days. Heat exchangers and precision mixers were used to control tank water temperature within 0.1°C and programmed and monitored using the Supervisory Control and Data Acquisition system (SCADA) at SeaSim. Experimental tanks were immersed within temperature controlled exterior insulating jackets of seawater kept at treatment temperatures (one jacket per treatment) and each tank fitted with a TC Direct PT‐100 temperature probe to maintain ramp rate and programmed temperatures. Filtered seawater was continuously supplied to the experimental system at an inflow rate of 0.8 L/min and each tank was fitted with a circulator (Turbelle nanostream 6015 3.5 W, Tunze Aquarientechnik Gmbh, Germany) for water movement. Light was on a 24 h 12:12 light/dark cycle with a 2‐h sunrise/sunset ramp time (half‐sine ramp) and maximum photosynthetically active radiation (PAR) at 280 μmol/m^2^/s (replicating the natural conditions of Davies Reef) supplied from SS Gen 1 Customized multichip LEDs (325 W, The National Sea Simulator, Townsville, Australia) and measured continuously in each tank using a Li‐192 sensor (UWQ8687, Li‐COR, USA) and Li‐250Am meter (Li‐COR, USA). Water temperature and PAR was stable at target levels throughout the experiment.

### 5.3 Sampling Plan

Corals were sampled at three time points (Figure 1): TP0, Day 0, at the start of the experiment (end of coral acclimation described above) but prior to temperature ramp‐up; TP1, Day 5, at the end of temperature ramp‐up; and TP2, Day 9, 4 days after the end of temperature ramp‐up. At each timepoint, sampling included photographs of each fragment (Figure S1) with one fragment per species from each tank (*n* = 3/treatment/species) immediately snap frozen and kept at −80°C until processing for metabolomics, proteomics, and algal endosymbiont cell counts and typing (see below). At each timepoint, and additionally, on Days 7 and 8, the maximal quantum yield (*F*_*v*_ */F*_*m*_) of chlorophyll a fluorescence of all fragments was measured using a Diving‐PAM (Waltz, Germany; Fiberoptics outer diameter 8 mm; settings: measuring light intensity = 6, saturation pulse intensity = 10, saturating width = 0.8 s, damping = 2, gain = 4). Measurements were made before dawn; therefore, colonies were low light acclimated (Suggett et al. 2022). Each fragment was measured three times, at separate parts of the fragment, and the average values were recorded. Analysis of variance (ANOVA) was utilized to assess the difference in *F*_*v*_ */F*_*m*_ for each coral species. Additionally, Tukey‐HSD test was performed to do a pairwise comparison between ambient and thermal stress at each timepoint.

### 5.4 Endosymbiont Counts

A small section of tissue (~1 cm^2^) from each frozen fragment collected at each timepoint (TP0, TP1, and TP2) was air‐brushed in PBS buffer, separated, and washed three times in filtered seawater. These cells were used to calculate endosymbiont abundance. Cell quantification was determined visually via a hemocytometer (Neubauer Hemocytometer, Fisher Scientific, Loughborough, UK) with eight replicate quadrats counted for each sample. Surface area measurements of each air‐brushed coral skeleton were conducted using the single wax dipping method (Veal et al. 2010). Eight cylindrical calibration standards were measured using digital calipers with a precision of 0.001 mm to determine their geometric surface areas. These were used to generate a standard curve relating wax mass to surface area. Coral skeletons were weighed, and then skeletons and calibration standards were immersed in paraffin wax at 65°C for 2 s, then removed and gently rotated to promote uniform coating and initial air drying. Samples were subsequently left to dry for an additional 15 min at ambient temperature before being reweighed. The surface area of each skeleton was then calculated based on the difference in mass before and after wax coating, using the calibration curve.

Endosymbiont cell densities were subsequently normalized using surface area, that is, cell density/surface area (cells/ml/cm^2^), hereinafter cell abundance. Normalized cell abundance were log_2_ transformed for visualization and additional statistical analysis. Prior to ANOVA, assumptions were verified using the Shapiro–Wilk test and Levene's test. Analysis of variance (ANOVA) was utilized to assess the difference in normalized cell density for each coral species. Additionally, Tukey‐HSD test was performed to do a pairwise comparison between ambient and thermal stress at each timepoint.

### 5.5 Endosymbiont Typing

A small section of tissue (~2 cm^2^) from each frozen fragment collected at TP0 was air‐brushed in PBS buffer for endosymbiont typing using amplicon sequencing of the ribosomal Internal Transcribed Spacer 2 (ITS‐2) region. The tissue slurry was centrifuged at 3000 g for 10 min to separate the coral host and algal symbiont fractions. DNA was extracted from the algal pellet using the Qiagen DNeasy Blood and Tissue kit (Qiagen, Germany) following the “protocol: purification of total DNA from animal tissues” including a RNase A‐based RNA digestion procedure. Genomic DNA was sent to the Ramaciotti Centre for Genomics (University of New South Wales, Sydney, Australia) and ITS‐2 amplicons sequenced using the sym_var_5.8 s2/sym_var_rev primers on the NextSeq 1000 P1 platform with 2 × 300bp paired‐end sequencing (including PhiX spike‐in) (Hume et al. 2018). ITS2 sequences were submitted to the SymPortal analytical framework for quality control and ITS2 type profile analysis as described by Hume and coauthors (Hume et al. 2019).

### 5.6 Protein Extraction

Proteins were extracted using a RIPA lysis buffer, composed of Tris–HCl (50 mM), NaCl (150 mM), SDS (1%), and DI water. The buffer was chilled on ice until ready for use and one complete mini EDTA‐free tablet was dissolved in 10 mL of lysis buffer immediately before extraction. A total of 500 μL of ice‐cold RIPA lysis buffer was added to a tube filled with 0.5 mm silica beads. Approximately 0.2–0.4 g of coral sample was clipped into each bead tube. The samples were vortexed for at least 5 min, or until the skeleton was stripped from the tissue. The samples were then incubated on ice for 30 min, before centrifuging at 10,000 rcf for 10 min and extracting the supernatant, while avoiding coral skeleton. Protein quality was assessed using a Qubit protein assay. Samples were stored in a freezer at −80°C.

### 5.7 Proteomics Analysis

Proteomic analysis was performed at the University of South Florida Proteomics Core. The proteins were normalized before digestion using 25 μg for each sample. After digestion, an equal amount of sample (1000 ng) was injected for each run. Instrument performance and stability were monitored using quality control (QA/QC) injections of pre‐digested HeLa cell extracts (Pierce, Cat. #88329), which were run before, during, and after sample batches and evaluated based on protein identifications, retention time stability, and signal intensity. LC–MS analysis peptides were characterized using a Thermo Q‐exactive‐HF‐X mass spectrometer coupled to a Thermo Easy nLC 1200. Samples were separated at 300 nL/min on an Acclaim PEPMAP 100 trap (75 μM, 2 cm, c18 3 μm, 100 A) and a Thermo easy spray column (75 μm, 25 cm, c18, 100 A) using a 180‐min gradient with an initial starting condition of 2% B buffer (0.1% formic acid in 90% Acetonitrile) and 98% A buffer (0.1% formic acid in water). Buffer B was increased to 28% over 140 min, then up to 40% in an additional 10 min. High B (90%) was run for 15 min afterwards. The mass spectrometer was outfitted with a Thermo nanospray easy source with the following parameters: Spray voltage: 2.00 V, Capillary temperature: 300 dC, Funnel RF level = 40. Parameters for data acquisition were as follows: for MS data the resolution was 60,000 with an AGC target of 3e^6^ and a max IT time of 50 ms, the range was set to 400–1600 m/z. MS/MS data was acquired with a resolution of 15,000, an AGC of 1e^4^, max IT of 50 ms, and the top 30 peaks were picked with an isolation window of 1.6 m/z, using a dynamic exclusion window of 25 s to prevent repeated fragmentation of high‐abundance precursor ions and increase proteome coverage.

For each of the three species, a comprehensive metaproteomic database was assembled based on the ITS2 results (Figure 2C) to aid protein identification. A proteomic database for *Porites lobata* was assembled from: (1) predicted genes from the *P. lobata* transcriptome assembly (v1) (Bhattacharya et al. 2016); (2) predicted genes from the *P. lobata* (KU572435) mitochondrial genome assembly (Tisthammer et al. 2016); (3) predicted genes from the *Cladocopium goreaui* nuclear genome assembly (Chen et al. 2022), (4) predicted genes from the *Breviolum minutum* mitochondrial genome assembly (Shoguchi et al. 2015); and (5) predicted genes from the *Cladocopium* C3 plastid genome assembly (Barbrook et al. 2014). A proteomic database for *Acropora hyacinthus* was assembled from: (1) predicted genes from the *A. hyacinthus* nuclear genome assembly (v1) (Singh et al. 2019); (2) predicted genes from the *A. hyacinthus* (OP311657) mitochondrial genome assembly (Wang et al. 2022); (3) predicted genes from the *Cladocopium goreaui* nuclear genome assembly; (4) predicted genes from the *Breviolum minutum* mitochondrial genome assembly; and (5) predicted genes from the *Cladocopium* C3 plastid genome assembly. Finally, a proteomic database for *Stylophora pistillata* was assembled from: (1) predicted genes from the *S. pistillata* nuclear genome assembly (v1) (Voolstra et al. 2017); (2) predicted genes from the *S. pistillata* (EU400214) mitochondrial genome assembly; (3) predicted genes from the *Cladocopium goreaui* nuclear genome assembly; (4) predicted genes from the *Breviolum minutum* mitochondrial genome assembly; and (5) predicted genes from the *Cladocopium* C3 plastid genome assembly.

### 5.8 Protein Clean‐Up and Normalization

Raw proteomic data was generated using Max quant 2.0.3.1 (Cox and Mann 2008; Tyanova et al. 2016). Proteomic data cleaning was conducted in RStudio v2024.04.2 + 764 using R v4.4.1 and the protti R package v0.9.0 (Quast et al. 2022). This package was used to clean, normalize, and generate pre‐ and post‐cleaning reports for each of the three host species. Assessments of peak intensity distribution, data completeness, coefficient of variation across sites, and principal component analysis (PCA) were performed before and after cleaning. To create a high‐quality protein group library, LC–MS results were filtered to exclude symbiont proteins, proteins with greater than one unique peptide and razor peptide for each protein group (Unique peptides > 1 & Razor + unique peptides > 1) and a protein group Q‐value less than 0.01. Proteins belonging to a set of potential contaminants provided by the LC–MS facility were also removed. The data matrix was then filtered to exclude observations with intensity values below 5, which likely represent false assignments. Protein intensities were log2‐transformed using the dplyr *mutate* and base R *log2* functions. Outlier samples were identified by PCA and data completeness using the protti *qc_pca* and *qc_data_completeness* functions. Based on these analyses, two samples with low completeness and that were PCA outliers in their respective datasets were removed, Acropora_638 and Stylophora_2312. Protein groups missing from more than 80% of samples were also removed. The 80% completeness threshold is typically used as a starting point for proteomic and metabolomic studies. We deemed after testing this and other thresholds that a missingness threshold of 80% effectively reduced technical noise without reducing biological signal. This threshold allowed proteins present in at least 80% of samples from a species to remain in the dataset, therefore it had to have been missing in > 3 samples to be removed. For the remaining high‐confidence observations, missing values were imputed using the *missForest* function from the missForest package with default parameters (maxiter = 10, ntree = 100) and a set seed of 124. Finally, log2‐transformed peak intensities were median‐normalized per treatment_timepoint group using the protti normalize function, resulting in the final cleaned protein abundance matrix.

### 5.9 Downstream Proteomic Analysis

GhostKOALA (v2.0) (Kanehisa et al. 2016) was used to annotate proteins with KEGG Orthology (KO) numbers. We subsequently calculated the log_2_ Fold‐Change (hereinafter, FC) for each protein between the ambient and high temperature samples for TP0, TP1, and TP2 using Python (v.3.9.12) (additional packages pandas v1.4.2 and numpy v1.26.4) and using SciPy (v1.12.0) to assess statistical significance with a student *t*‐test (*n* = 3). A protein was considered to have an increased abundance if it had a FC > 0.5 and *p*‐value < 0.05; likewise, a protein was considered to have a decreased abundance if it had a FC < −0.5 and *p*‐value < 0.05. However, upon correcting for multiple testing (Benjamini Hochberg corrections), no adjusted *p‐*value was < 0.05, potentially due to the high variation in the proteomic data. We therefore report unadjusted protein‐level *p*‐values and use the pathways analysis to support our conclusions, since it is less likely that a Type I error will occur in our analysis if all proteins in a pathway support a change in activity (see below). We explicitly acknowledge this limitation in the Study Limitations section.

We also performed a pathway level analysis, using the “B‐level Description” annotation generated from the KEGG database (http://www.genome.jp/kegg‐bin/get_htext?ko00001.keg). Pathway‐level responses were assessed using a percentage‐based metric rather than conventional enrichment approaches. Traditional pathway enrichment tools, such as Gene Set Enrichment Analysis (GSEA), require broad proteome coverage across pathway members to generate robust statistical inferences. In this dataset, proteome coverage was limited across species (~9.7% of *A. hyacinthus proteins had proteomic data*, ~5.1% of *S. pistillata proteins*, and ~11.1% of *P. lobata* proteins), precluding reliable use of abundance‐based enrichment methods. We therefore evaluated pathway responses based on the proportion of detected proteins within each pathway exhibiting directional changes in abundance. Pathways were classified as upregulated when ≥ 5% of detected proteins showed increased abundance under thermal stress and downregulated when ≥ 5% showed decreased abundance. This threshold was selected to enable detection of coordinated pathway‐level trends while minimizing the influence of individual protein‐level variability.

### 5.10 Metabolomic Analysis

In addition to physiological and proteomic analyses, we generated untargeted polar metabolomics data to assess metabolite abundance (notably dipeptides) previously linked to thermal stress responses in corals (Williams, Pathmanathan, et al. 2021). Metabolites were extracted from preserved coral tissue using a 40:40:20 methanol:acetonitrile:water buffer with 0.1 M formic acid, followed by homogenization, centrifugation, acid neutralization with ammonium bicarbonate, and transfer to autosampler vials for LC–MS analysis. LC–MS analysis was performed at the Metabolomics Core Facility at the Cancer Institute of New Jersey. Prior to sample runs, LC–MS performance was verified using commercial and in‐house standards to ensure mass accuracy and signal consistency. Samples were randomized to minimize batch effects, and method blanks were included to assess background signals. Metabolite separation was performed using HILIC on a Vanquish UHPLC (Thermo Fisher Scientific, Waltham, MA) and the subsequent full scan mass spectrometry was performed on the Thermo Q Exactive PLUS mass spectrometer operating in both positive and negative modes. Data were processed using MAVEN (Melamud et al. 2010) with identification based on accurate mass and retention time matching to an in‐house library, and low‐abundance metabolites were filtered from downstream analyses. For an in‐depth description of the extraction protocols and mass‐spectrometry settings (see Data S1).

We subsequently calculated the log_2_FC for each of the metabolites across the species, comparing the ambient and thermally stressed treatments at each timepoint. We also assessed the statistical significance for each of the metabolites using a Welch *t*‐test (*n* = 6) with multiple testing correction using the Benjamini‐Hochberg procedure, using SciPy (v1.12.0).

## Author Contributions

**Shrinivas Nandi:** conceptualization (equal), formal analysis (equal), investigation (equal), methodology (equal), visualization (equal), writing – original draft (equal), writing – review and editing (equal). **Timothy G. Stephens:** conceptualization (equal), formal analysis (equal), investigation (equal), methodology (equal), visualization (equal), writing – original draft (equal), writing – review and editing (equal). **Erin E. Chille:** conceptualization (equal), formal analysis (equal), investigation (equal), methodology (equal), visualization (supporting), writing – original draft (equal), writing – review and editing (supporting). **Samantha Goyen:** conceptualization (equal), data curation (equal), formal analysis (supporting), methodology (equal), visualization (supporting), writing – review and editing (equal). **Line K. Bay:** conceptualization (equal), funding acquisition (equal), resources (equal), supervision (equal), writing – review and editing (equal). **Debashish Bhattacharya:** conceptualization (equal), funding acquisition (equal), investigation (equal), methodology (equal), resources (lead), supervision (lead), writing – original draft (equal), writing – review and editing (equal).

## Funding

National Science Foundation award titled “Collaborative Research: Edge CMT: Polygenic traits of heat stress phenome in coral” dark genes “from genome to functional applications” (#2128073; 12/01/2021 to 11/30/2025) awarded to D.B., USDA National Institute of Food and Agriculture Hatch Formula award titled “Using multi‐omics and experimental evolution to understand the response of eukaryotes to changing environmental conditions” (#NJ01180; 01/17/20 to 09/30/24) awarded to DB, Revive & Restore award titled “Toward predictive coral phenomics” (#2022–039; 01/10/2022 to 09/30/2025) awarded to L.K.B., S.G., and D.B., and the Australian Government Reef Trust and Great Barrier Reef Foundation titled “Reef Restoration and Adaptation Program (RRAP)” awarded to L.K.B.

## Conflicts of Interest

The authors declare no conflicts of interest.

## Supporting information


**Figure S1:** Coral fragment images over the course of the experiment. Coral fragments from all three species at various timepoints during the experiment. *S. pistillata* showed distinct paling at TP1 under thermal stress. Similarly, *A. hyacinthus* displays clear signs of bleaching by TP2 based on visual assessment. In *P. lobata* , mild paling is also evident by TP2. Timepoints and colony IDs have been presented in the figure.

**Figure S2:** Host total proteome distribution. Volcano plots summarizing host proteome responses across species and time points. Proteins are arranged by species (horizontal) and time point (vertical); data are not available for *S. pistillata* at TP2 due to the bailout phenotype (indicated as “NA”). Statistical thresholds are defined in the figure key.

**Figure S3:** Fold‐change based pathway level assessment. Fold‐change‐based pathway‐level assessment of differentially abundant proteins at TP1 (A) and TP2 (B). Stacked mirror plots show the number of differentially abundant proteins per KEGG pathway (B‐description) across species; only pathways with more than five differentially abundant proteins are displayed. TP2 data are available only for *A. hyacinthus* and *P. lobata* .

**File S1:** Endosymbiont proteome distribution after cell density normalization and principal component analysis. Boxplots show log_2_‐transformed protein intensities across treatments and time points for *A. hyacinthus* (A), *P. lobata* (C), and *S. pistillata* (E) symbiont proteomes after group‐median normalization by treatment and time point. PCA plots display the first two principal components derived from normalized protein intensities for each species (B, D, F). Color and symbol definitions are provided in the figure keys.

**File S2:** Host protein intensity distribution and principal component analysis. Boxplots show log_2_‐transformed protein intensities across treatments and time points for *A. hyacinthus* (A), *P. lobata* (C), and *S. pistillata* (E) host proteomes after group‐median normalization by treatment and time point. PCA plots display the first two principal components derived from normalized protein intensities for each species (B, D, F). Color and symbol definitions are provided in the figure keys.

**Table S1:** (a) Endosymbiont cell density and photosynthetic efficiency for samples. (b) SymPortal ITS2 Symbiodiniaceae communities within *A. hyacinthus, P* *. lobata* *and S* *. pistillata* . ITS2 sequence data and major ITS2‐type profiles.

**Table S2:** Proteomic data for the *Acropora hyacinthus* host across timepoints, with differential accumulation statistics and KEGG annotations.

**Table S3:** Proteomic data for the *Porites lobata* host across timepoints, with differential accumulation statistics and KEGG annotations.

**Table S4:** Proteomic data for the *Stylophora pistillata* host across timepoints, with differential accumulation statistics and KEGG annotations.

**Table S5:** Summary table of all GBR coral host proteins detected for each species across the stress experiment for specific pathways. Each protein is counted only once here for reference. Multiple copies of a given protein are included in these counts.

**Table S6:** (a) Dipeptide metabolite abundance variation between control and treatment conditions across timepoints. (b) Annotated antioxidant metabolite abundance variation between control and treatment conditions across timepoints. (c) Amino acid metabolite abundance variation between control and treatment conditions across timepoints.

**Data S1:** Supporting information.

## Data Availability

All data are available in the main text or the supporting materials. The GBR coral mass spectrometry proteomics data have been deposited to the ProteomeXchange Consortium via the PRIDE partner repository with the dataset identifier PXD052478 (https://www.ebi.ac.uk/pride/). The metabolomic mass spectrometry data are available from the MassIVE database with the dataset identifier MSV000094832 (https://massive.ucsd.edu/). The ITS2 sequence read files are available from the Sequencing Read Archive (SRA) with the dataset identifier PRJNA1119670 (https://www.ncbi.nlm.nih.gov/sra/).
