# Supplementary figures and images for: Metaproteome Analysis of Short‐Term Thermal Stress in Three Sympatric Coral Species Reveals Divergent Host Responses
Source: Ecol Evol. 2026 Mar 19;16(3):e73275. doi: 10.1002/ece3.73275 (PMC13093290; doi:10.1002/ece3.73275)

## *S. pistillata*

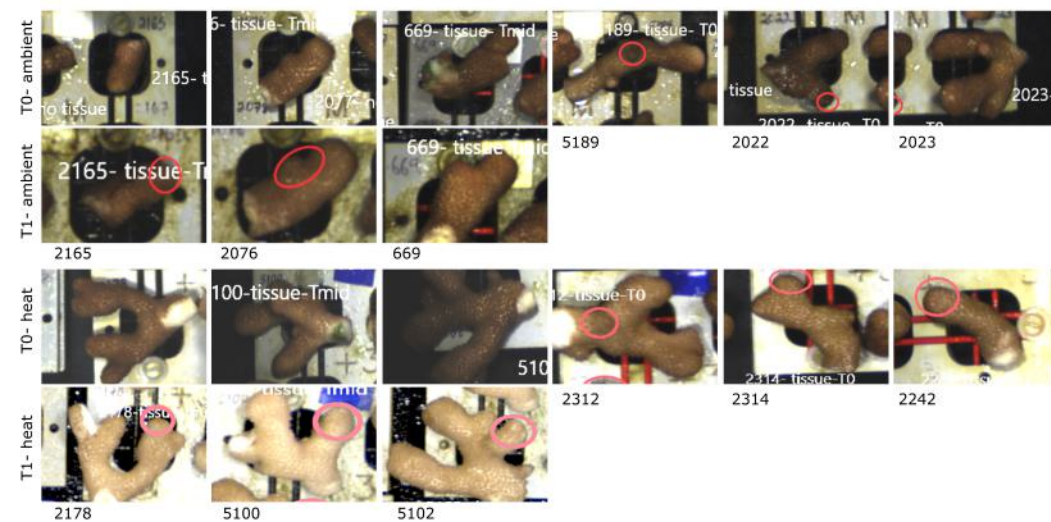

## *A. hyacinthus*

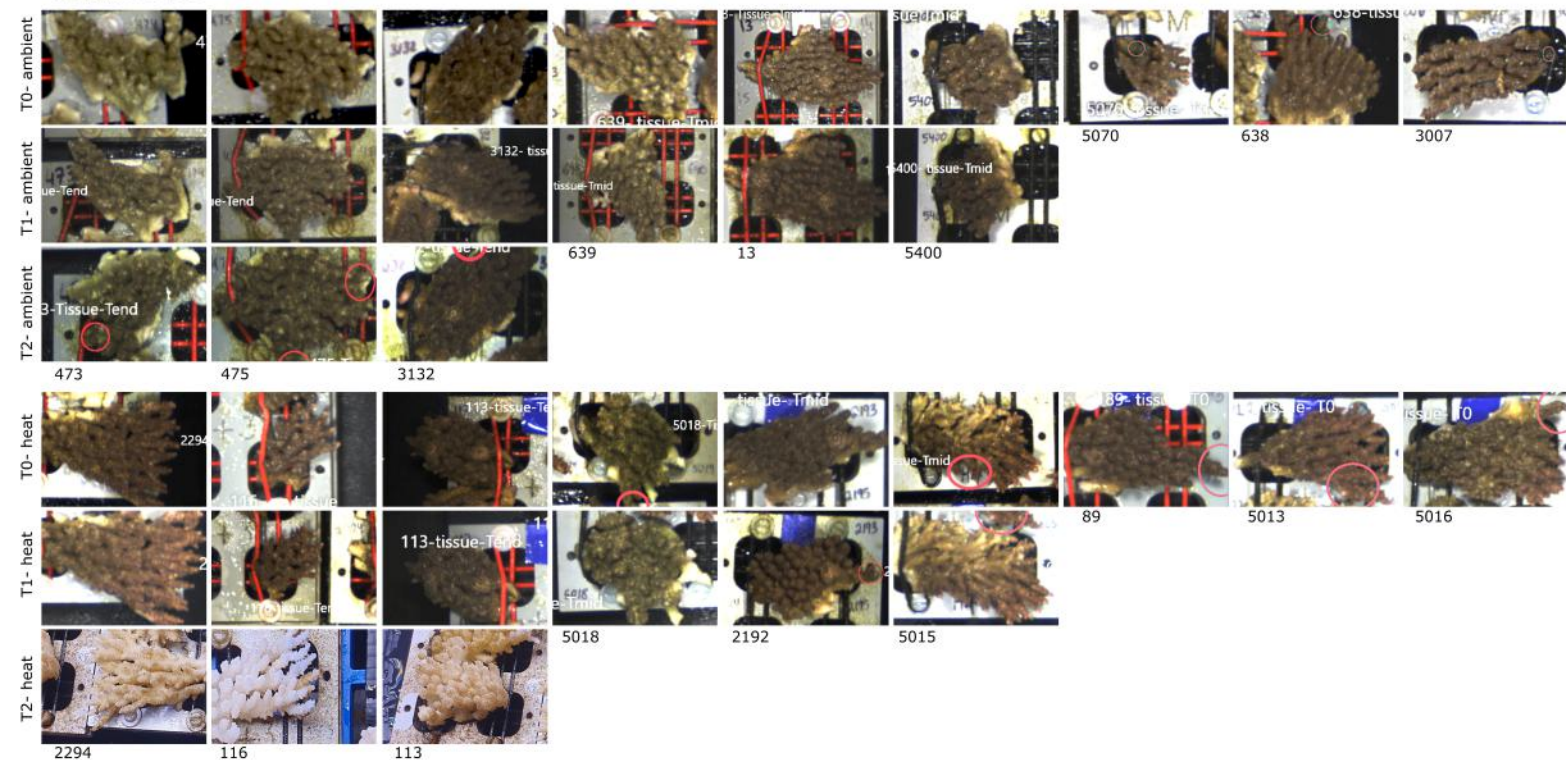

## *P. lobata*

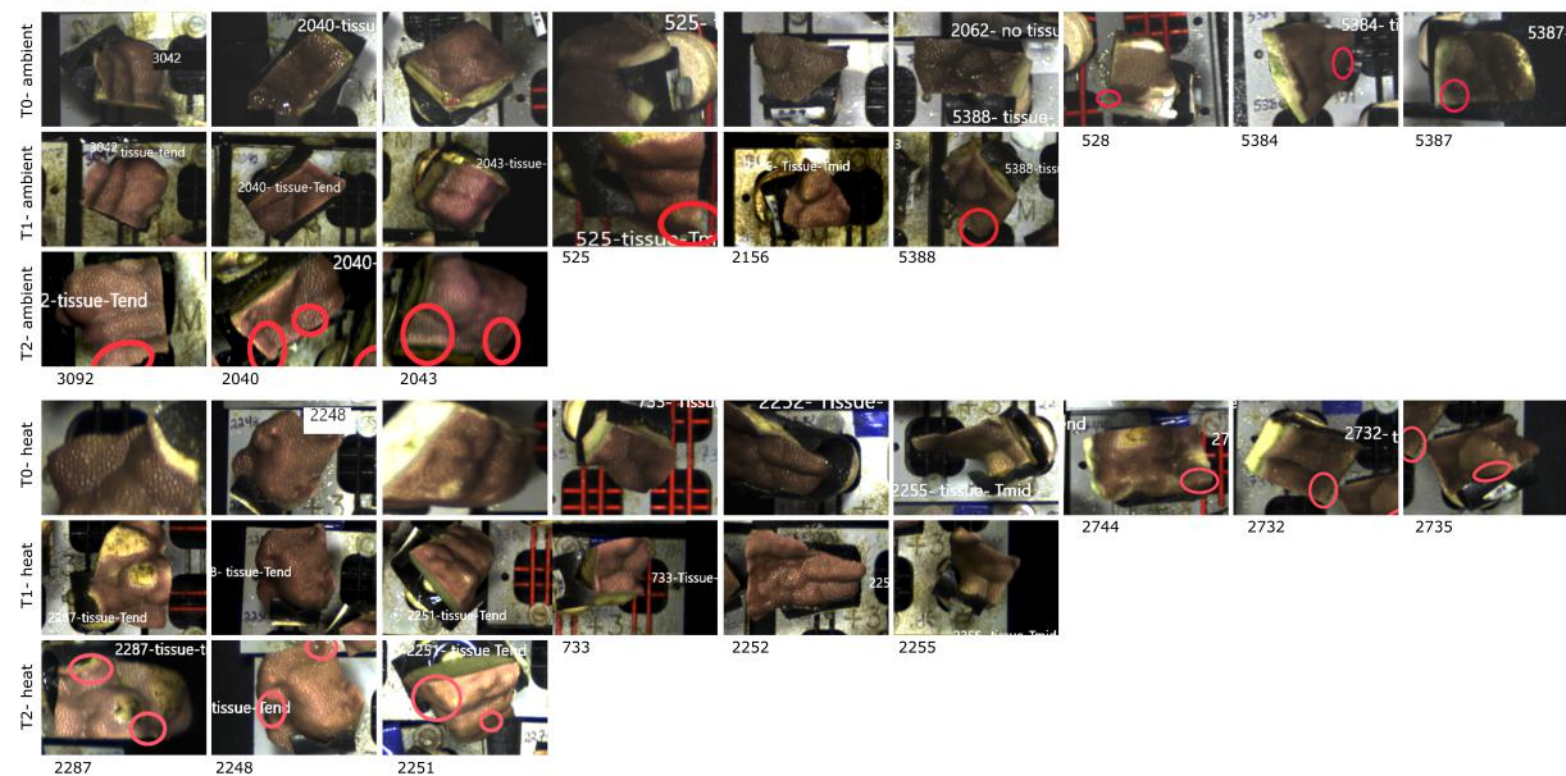

Supplement: Supplementary file 1 — Figure S1: Coral fragment images over the course of the experiment. Coral fragments from all three species at various timepoints during the experiment. S. pistillata showed distinct paling at TP1 under thermal stress. Similarly, A. hyacinthus displays clear signs of bleaching by TP2 based on visual assessment. In P. lobata , mild paling is also evident by TP2. Timepoints and colony IDs have been presented in the figure. [file ECE3-16-e73275-s012.pdf]

# Coral Proteomic Differential Abundance

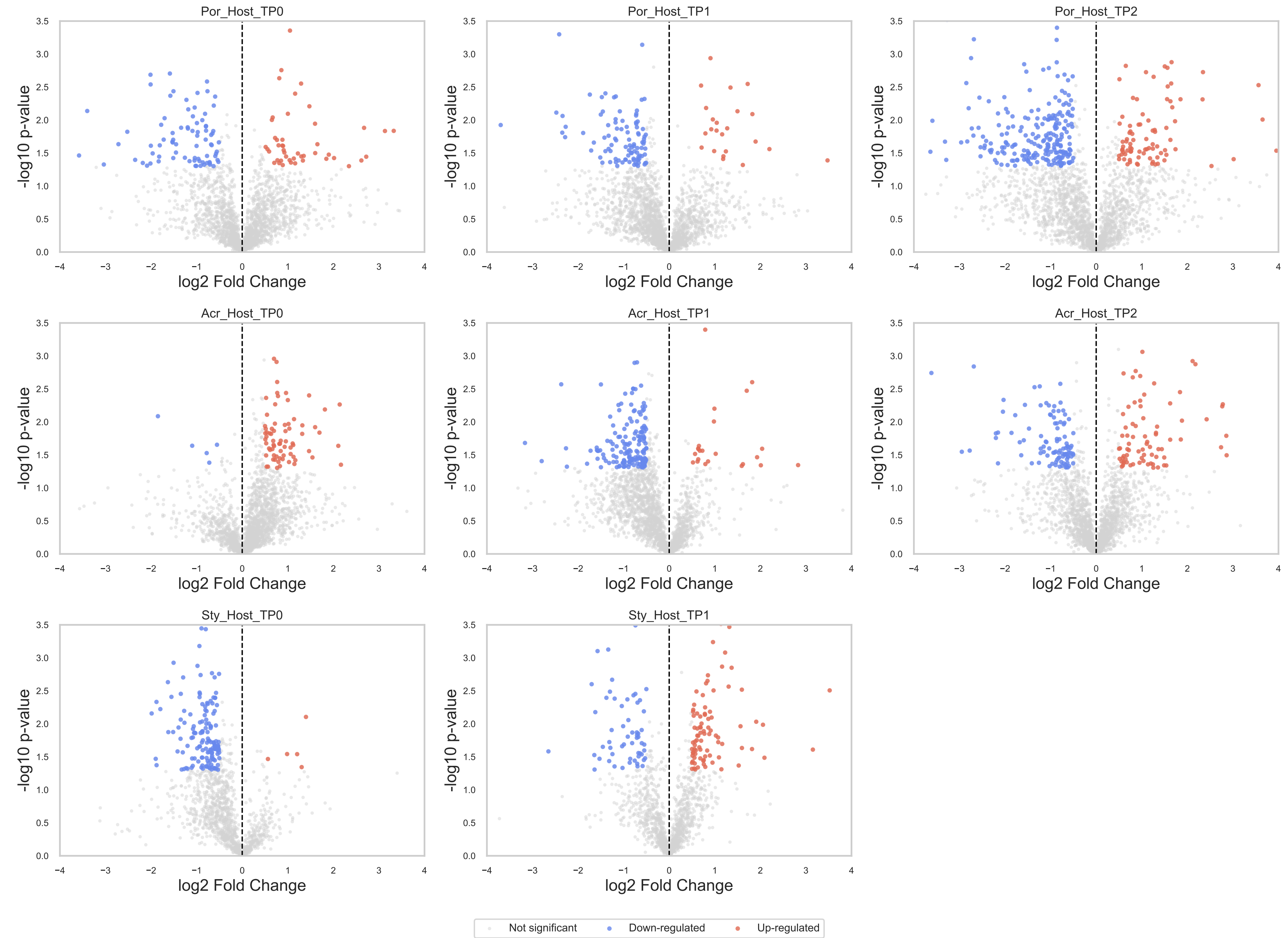

Supplement: Supplementary file 2 — Figure S2: Host total proteome distribution. Volcano plots summarizing host proteome responses across species and time points. Proteins are arranged by species (horizontal) and time point (vertical); data are not available for S. pistillata at TP2 due to the bailout phenotype (indicated as “NA”). Statistical thresholds are defined in the figure key. [file ECE3-16-e73275-s011.pdf]

Differentially Abundant Proteins Timepoint 1

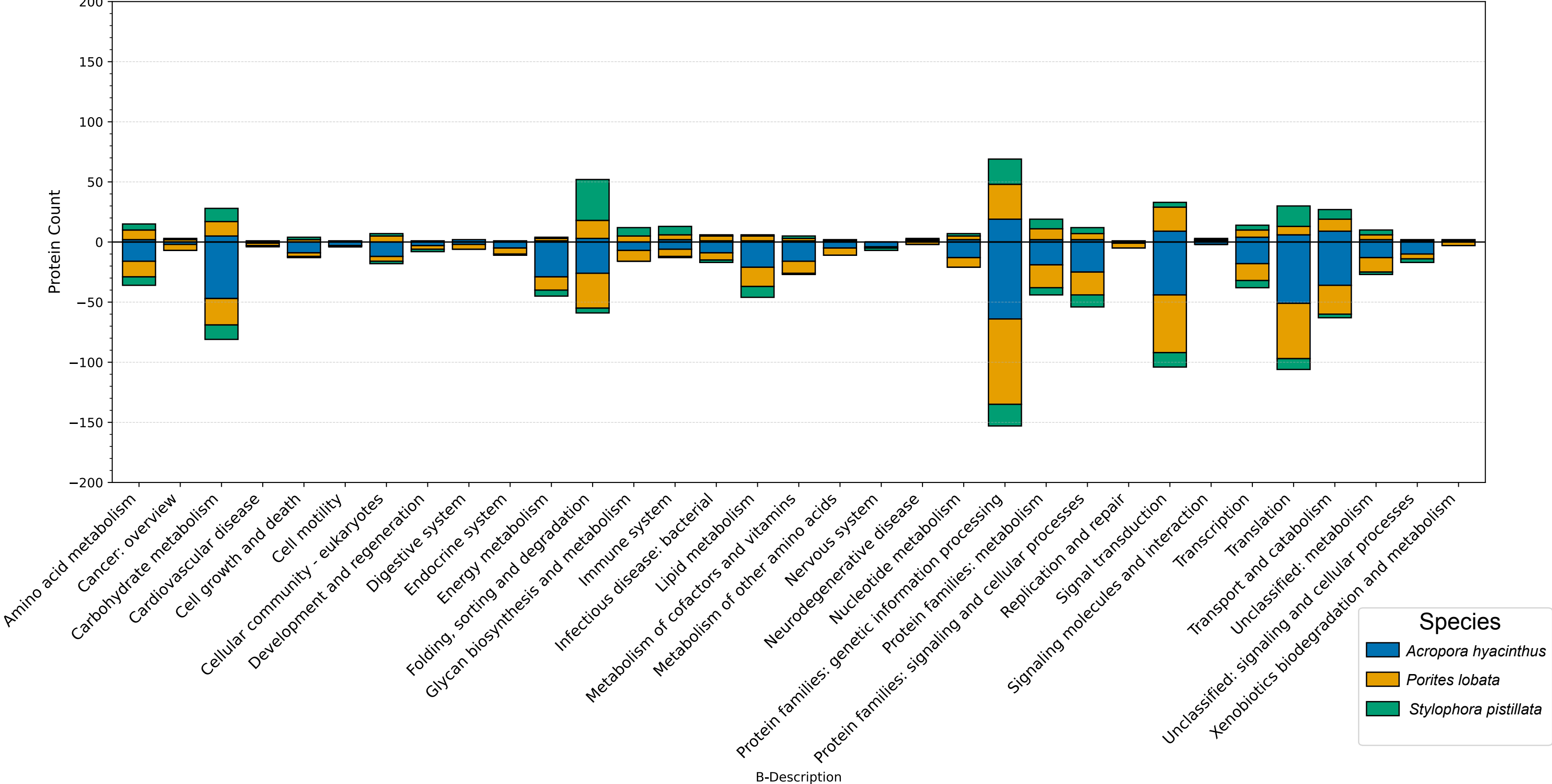

Differentially Abundant Proteins Timepoint 2

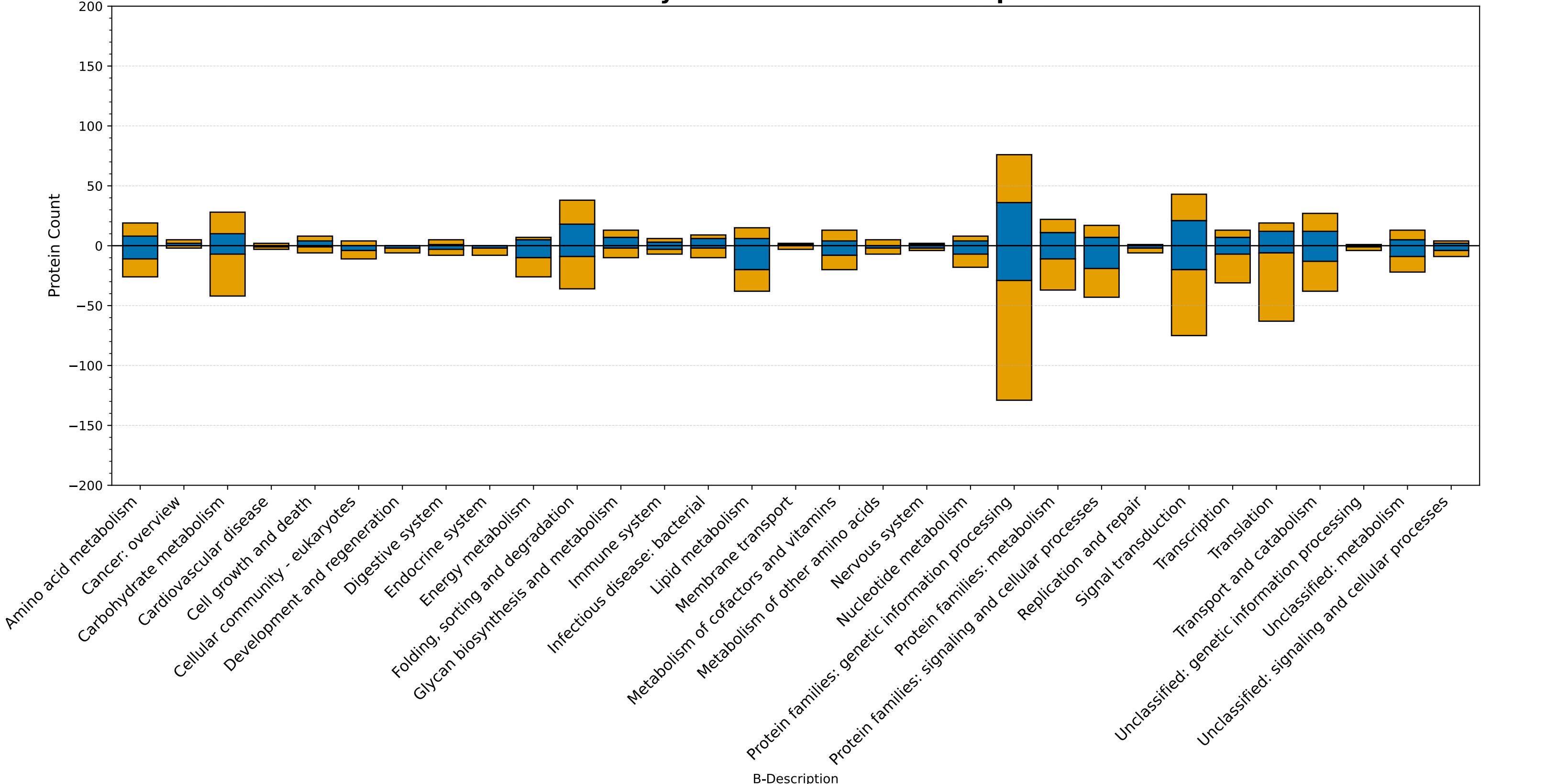

Supplement: Supplementary file 3 — Figure S3: Fold‐change based pathway level assessment. Fold‐change‐based pathway‐level assessment of differentially abundant proteins at TP1 (A) and TP2 (B). Stacked mirror plots show the number of differentially abundant proteins per KEGG pathway (B‐description) across species; only pathways with more than five differentially abundant proteins are displayed. TP2 data are available only for A. hyacinthus and P. lobata . [file ECE3-16-e73275-s003.pdf]
