# Supplementary material for: Metaproteome Analysis of Short‐Term Thermal Stress in Three Sympatric Coral Species Reveals Divergent Host Responses
Source: Ecol Evol. 2026 Mar 19;16(3):e73275. doi: 10.1002/ece3.73275 (PMC13093290; doi:10.1002/ece3.73275)
Supplement: Supplementary file 4 — File S1: Endosymbiont proteome distribution after cell density normalization and principal component analysis. Boxplots show log2‐transformed protein intensities across treatments and time points for A. hyacinthus (A), P. lobata (C), and S. pistillata (E) symbiont proteomes after group‐median normalization by treatment and time point. PCA plots display the first two principal components derived from normalized protein intensities for each species (B, D, F). Color and symbol definitions are provided in the figure keys. [file ECE3-16-e73275-s009.pdf]

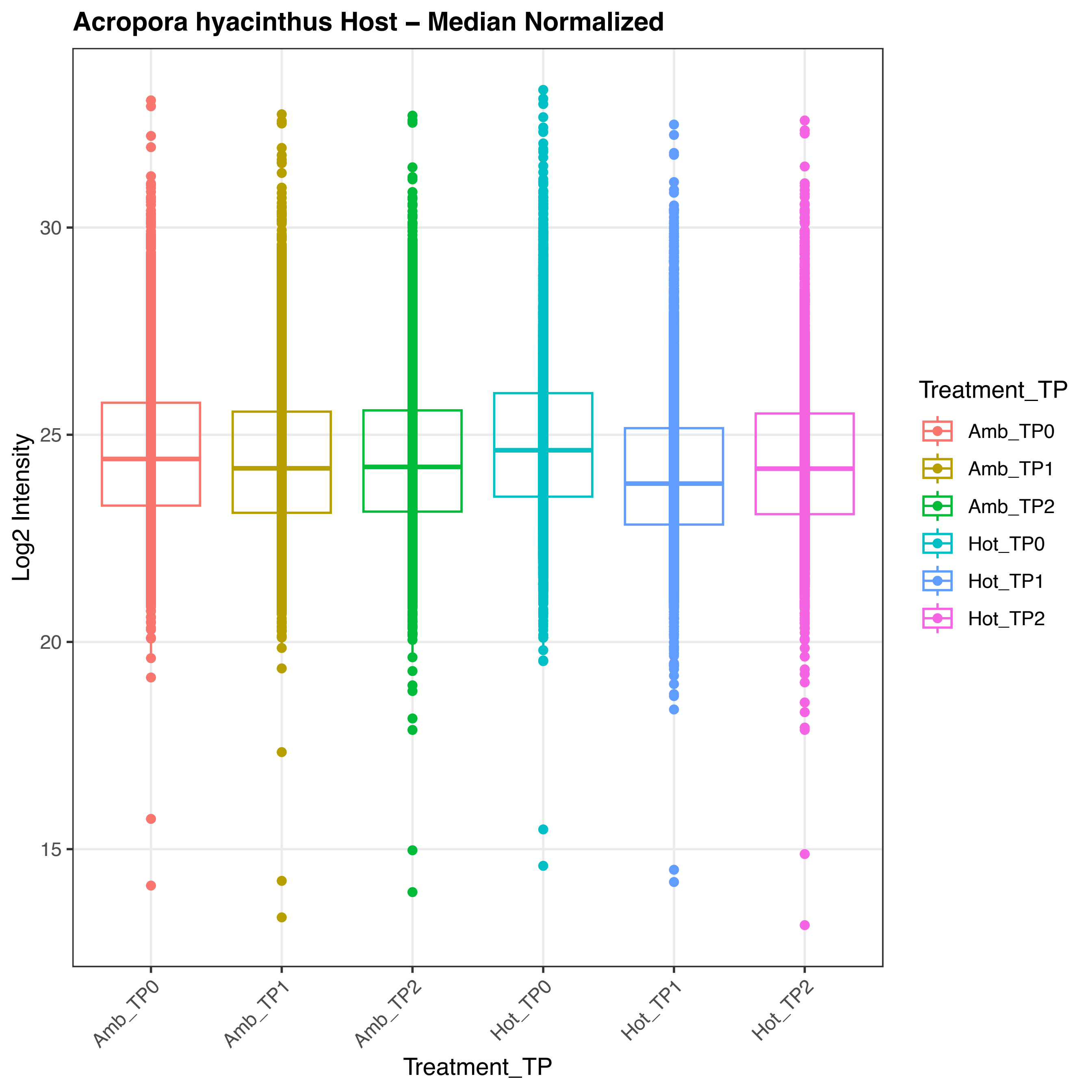
Acropora hyacinthus Host – Median Normalized
Log2 Intensity
30
25
20
15
Amb_TP0
Amb_TP1
Amb_TP2
Hot_TP0
Hot_TP1
Hot_TP2
Treatment_TP
Treatment_TP
Amb_TP0
Amb_TP1
Amb_TP2
Hot_TP0
Hot_TP1
Hot_TP2

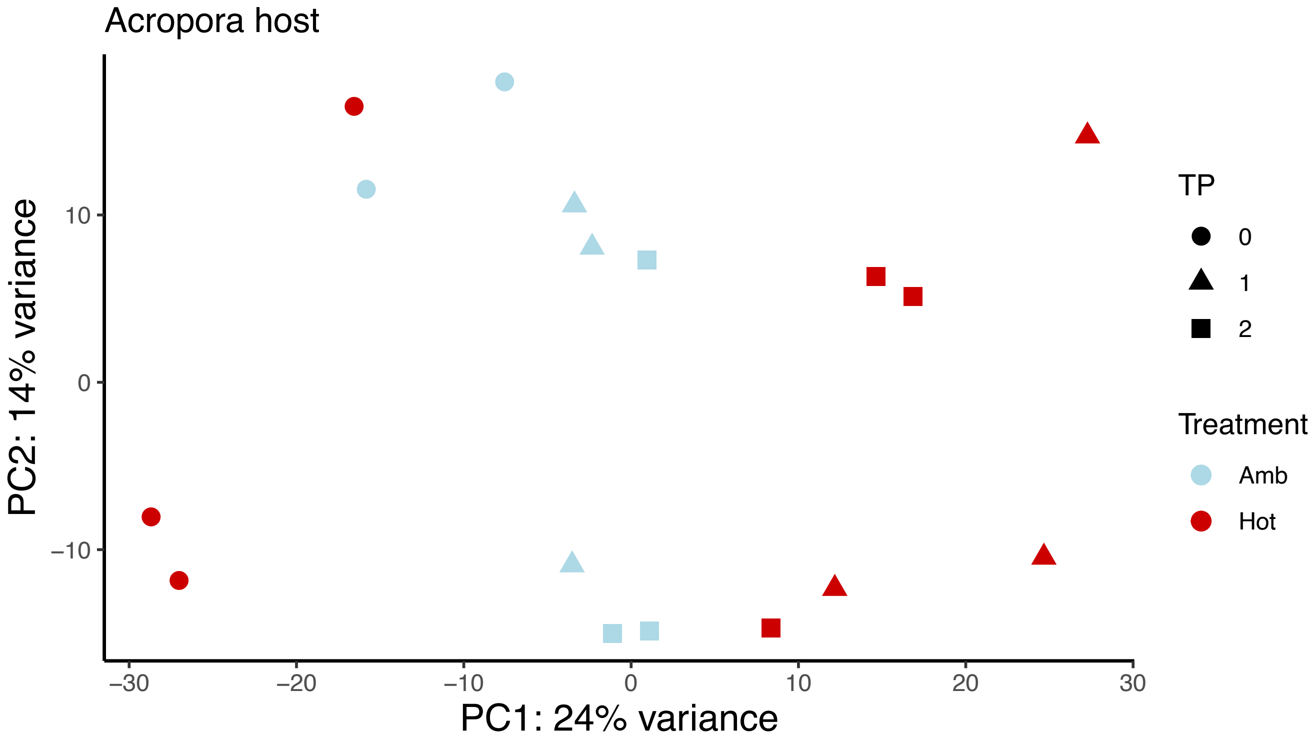

Acropora host
PC2: 14% variance
PC1: 24% variance
10
0
−10
−30
−20
−10
0
10
20
30
TP
0
1
2
Treatment
Amb
Hot

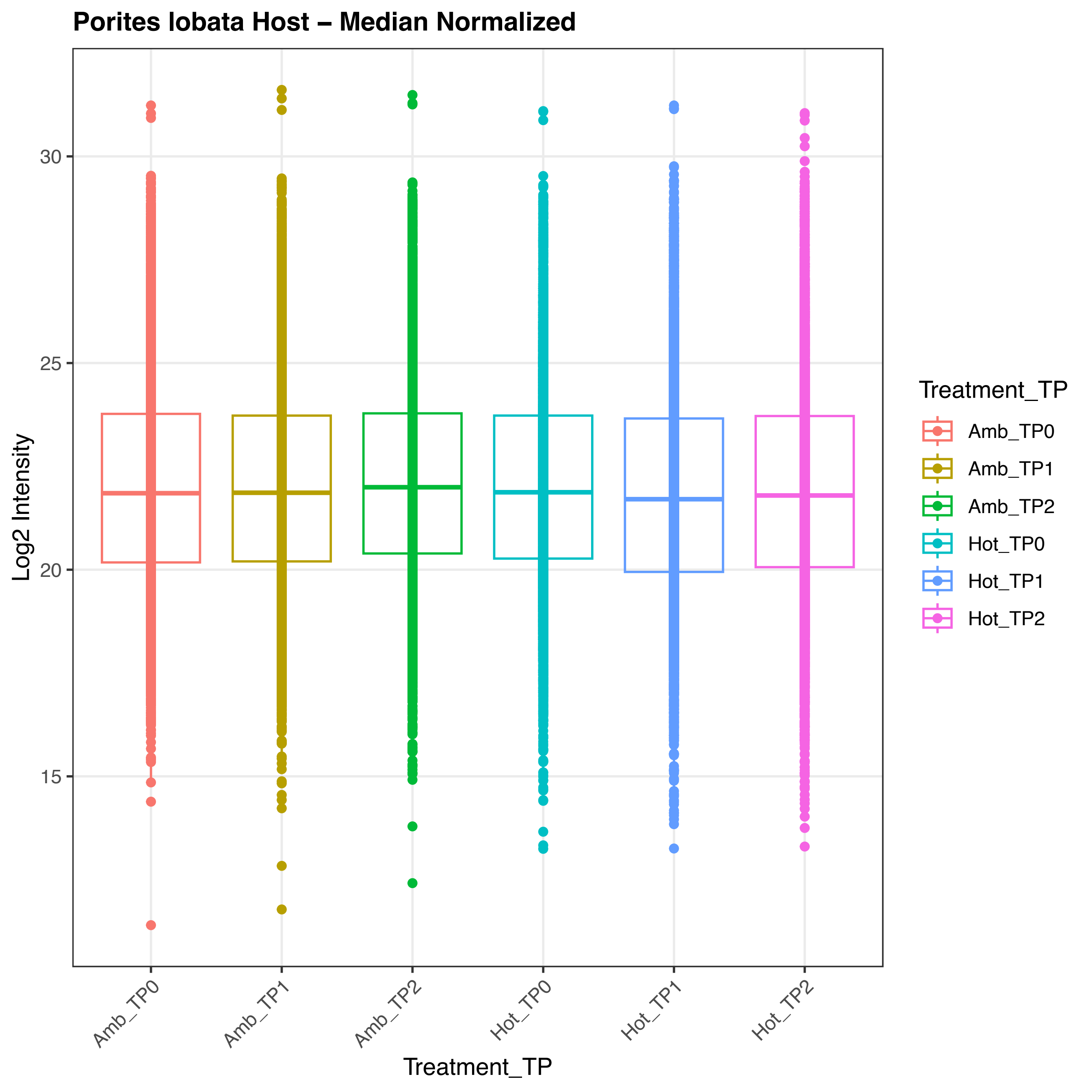

Porites lobata Host – Median Normalized
Log2 Intensity
30
25
20
15
Amb_TP0
Amb_TP1
Amb_TP2
Hot_TP0
Hot_TP1
Hot_TP2
Treatment_TP
Treatment_TP
Amb_TP0
Amb_TP1
Amb_TP2
Hot_TP0
Hot_TP1
Hot_TP2

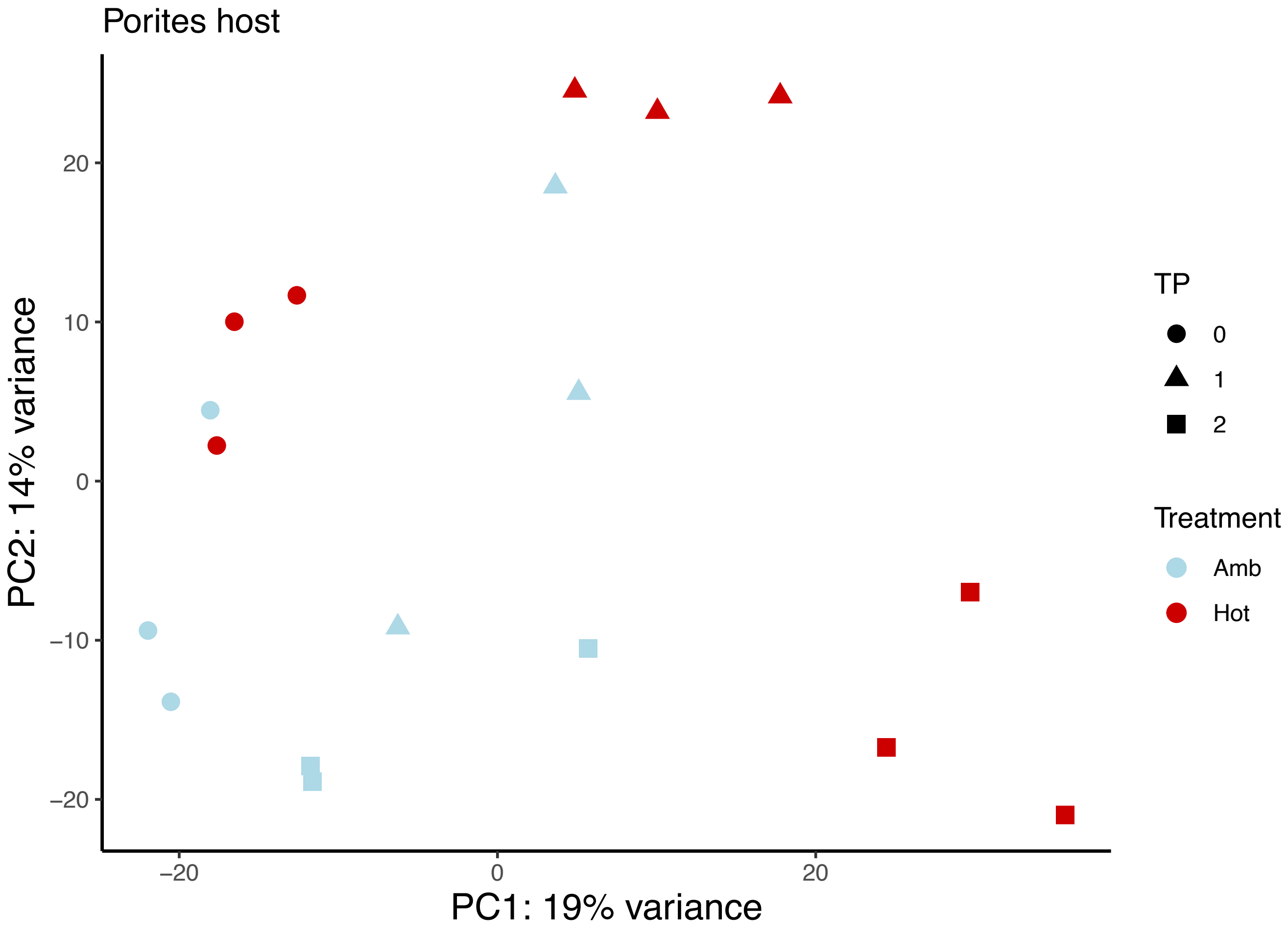

Porites host
PC2: 14% variance
PC1: 19% variance
20
10
0
−10
−20
−20
0
20
TP
0
1
2
Treatment
Amb
Hot

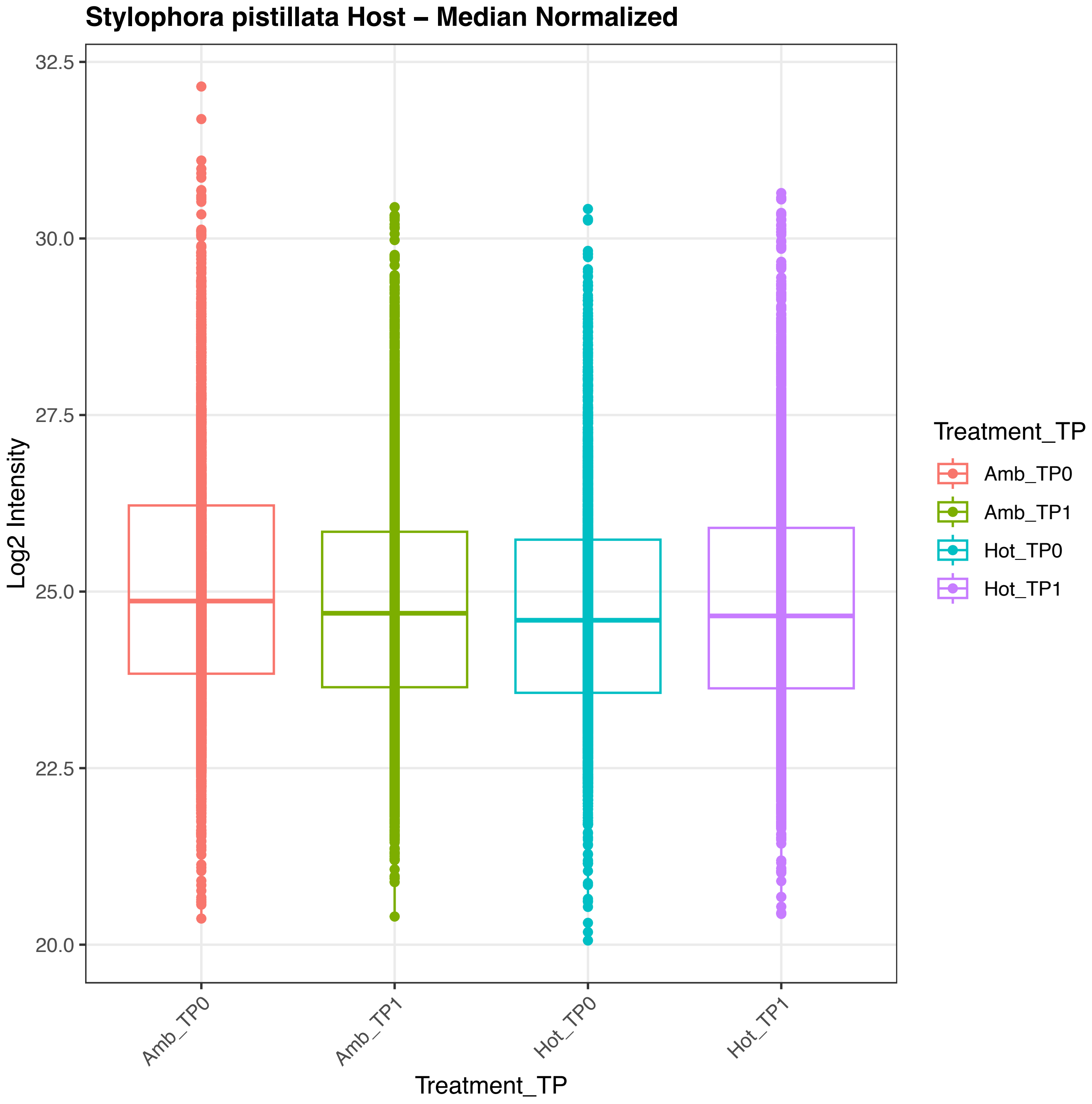
Stylophora pistillata Host – Median Normalized
Log2 Intensity
32.5
30.0
27.5
25.0
22.5
20.0
Amb_TP0
Amb_TP1
Hot_TP0
Hot_TP1
Treatment_TP
Treatment_TP
Amb_TP0
Amb_TP1
Hot_TP0
Hot_TP1

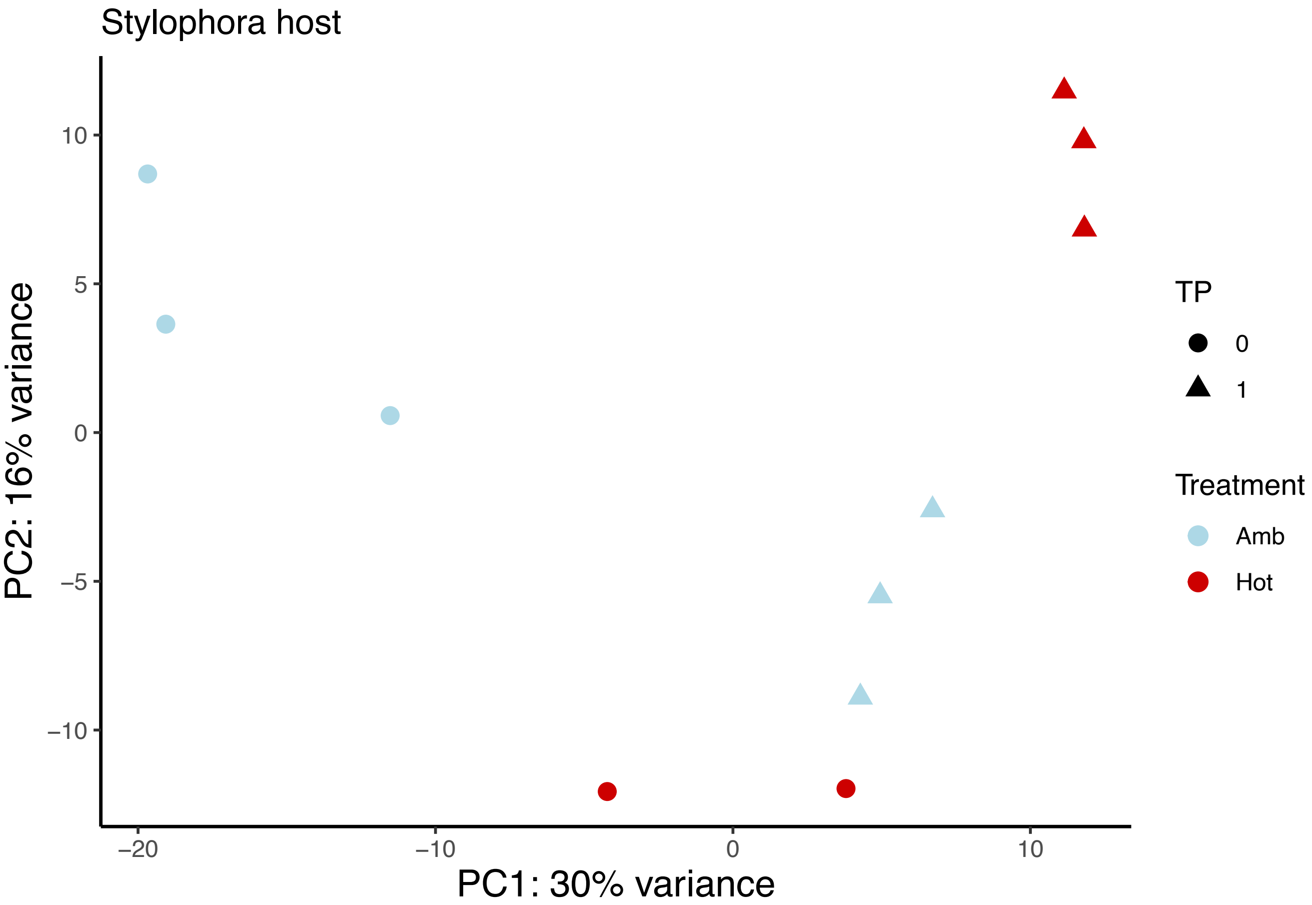

Stylophora host
PC1: 30% variance
PC2: 16% variance
−20
−10
0
10
5
−5
TP
0
1
Treatment
Amb
Hot
