# Supplementary material for: Metaproteome Analysis of Short‐Term Thermal Stress in Three Sympatric Coral Species Reveals Divergent Host Responses
Source: Ecol Evol. 2026 Mar 19;16(3):e73275. doi: 10.1002/ece3.73275 (PMC13093290; doi:10.1002/ece3.73275)
Supplement: Supplementary file 5 — File S2: Host protein intensity distribution and principal component analysis. Boxplots show log2‐transformed protein intensities across treatments and time points for A. hyacinthus (A), P. lobata (C), and S. pistillata (E) host proteomes after group‐median normalization by treatment and time point. PCA plots display the first two principal components derived from normalized protein intensities for each species (B, D, F). Color and symbol definitions are provided in the figure keys. [file ECE3-16-e73275-s004.pdf]

Acropora hyacinthus Symbiont – Group Median Normalized

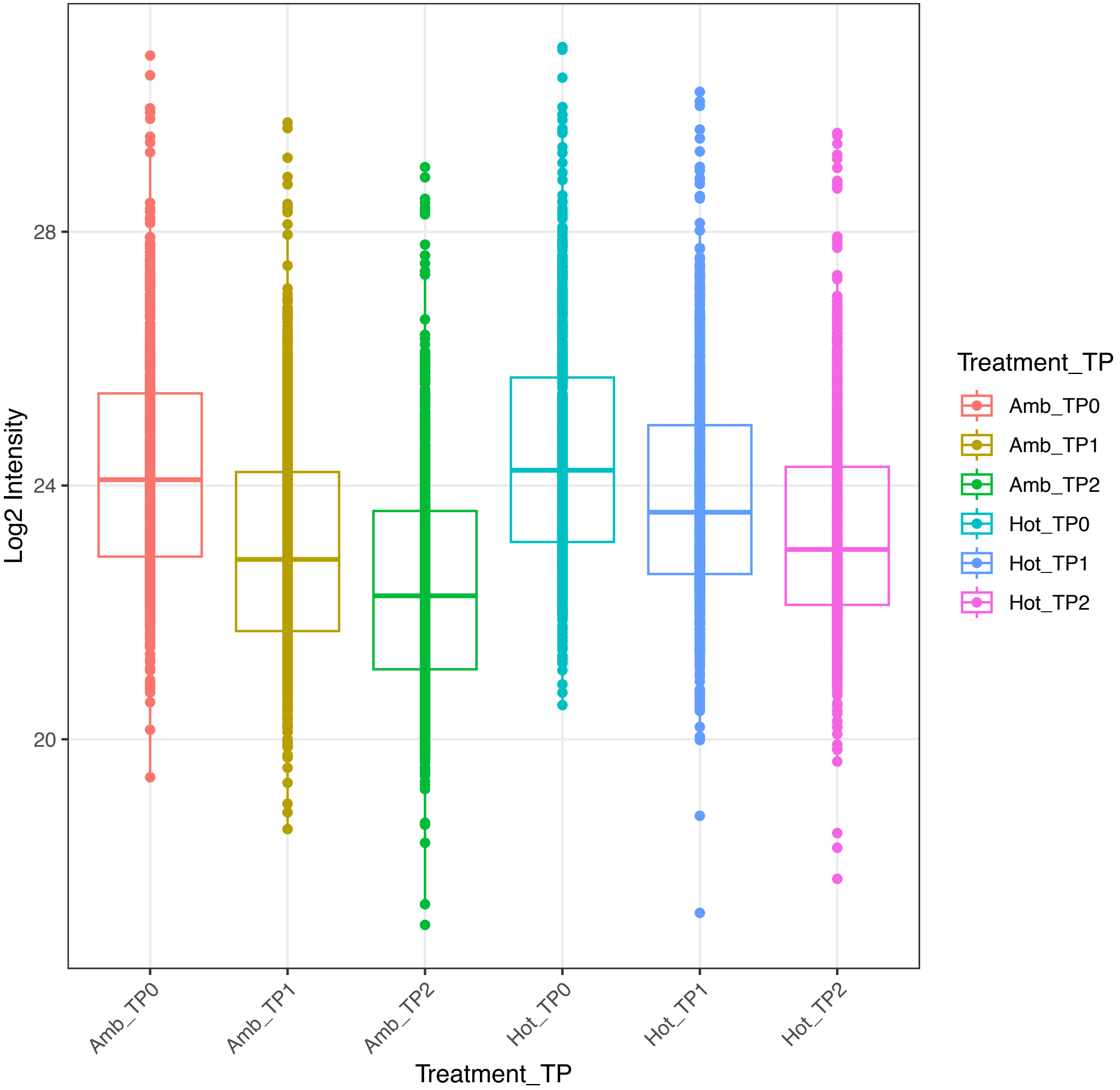

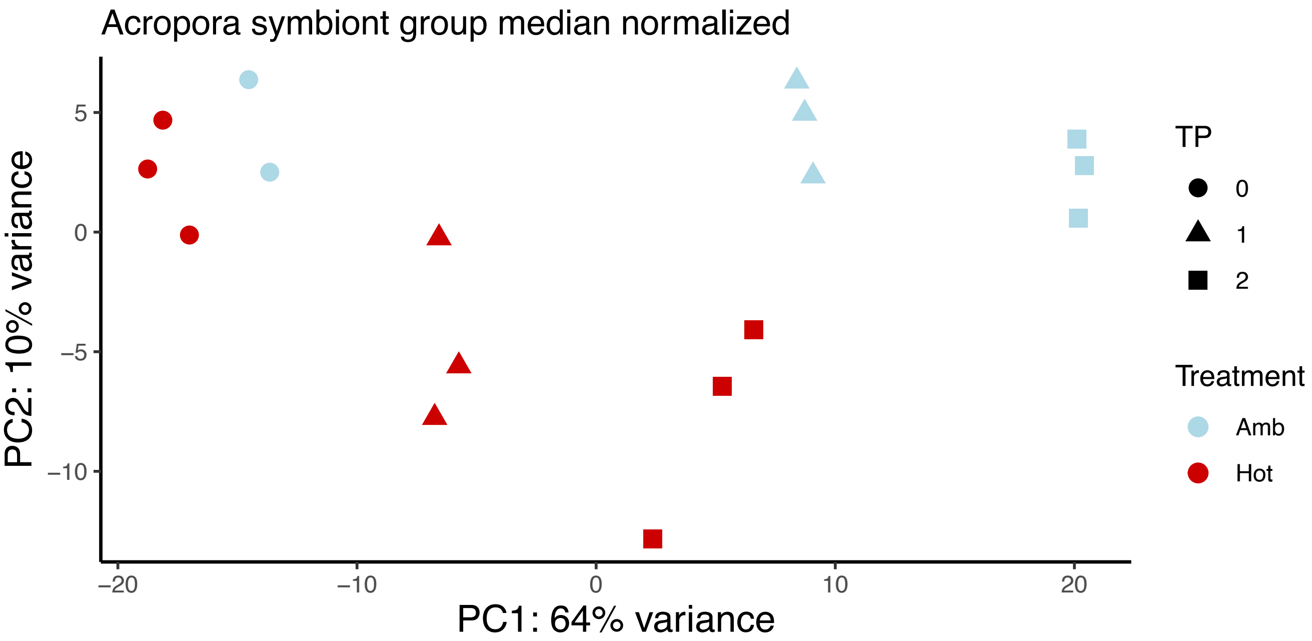

Acropora symbiont group median normalized
PC2: 10% variance
PC1: 64% variance
5
0
−5
−10
−20
−10
0
10
20
TP
0
1
2
Treatment
Amb
Hot

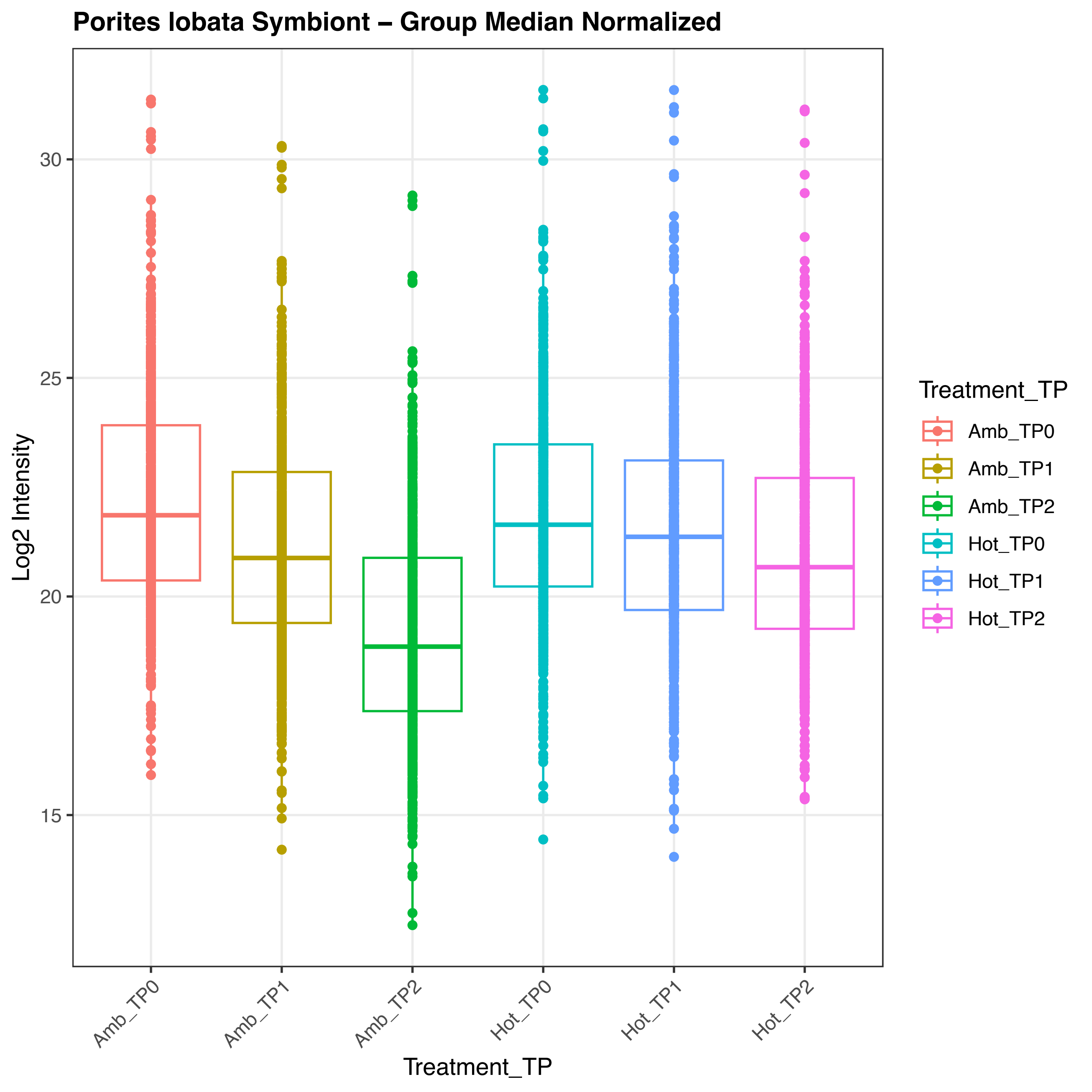

Porites lobata Symbiont – Group Median Normalized
Log2 Intensity
30
25
20
15
Treatment_TP
Amb_TP0
Amb_TP1
Amb_TP2
Hot_TP0
Hot_TP1
Hot_TP2

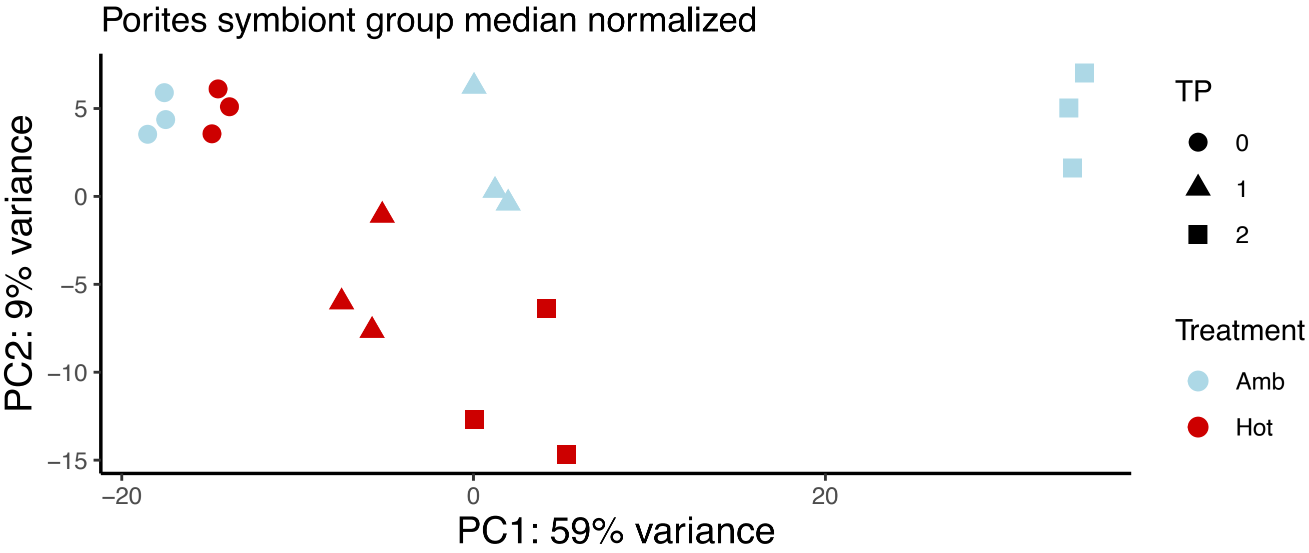

Porites symbiont group median normalized
PC2: 9% variance
PC1: 59% variance
5
0
−5
−10
−15
−20
0
20
TP
0
1
2
Treatment
Amb
Hot

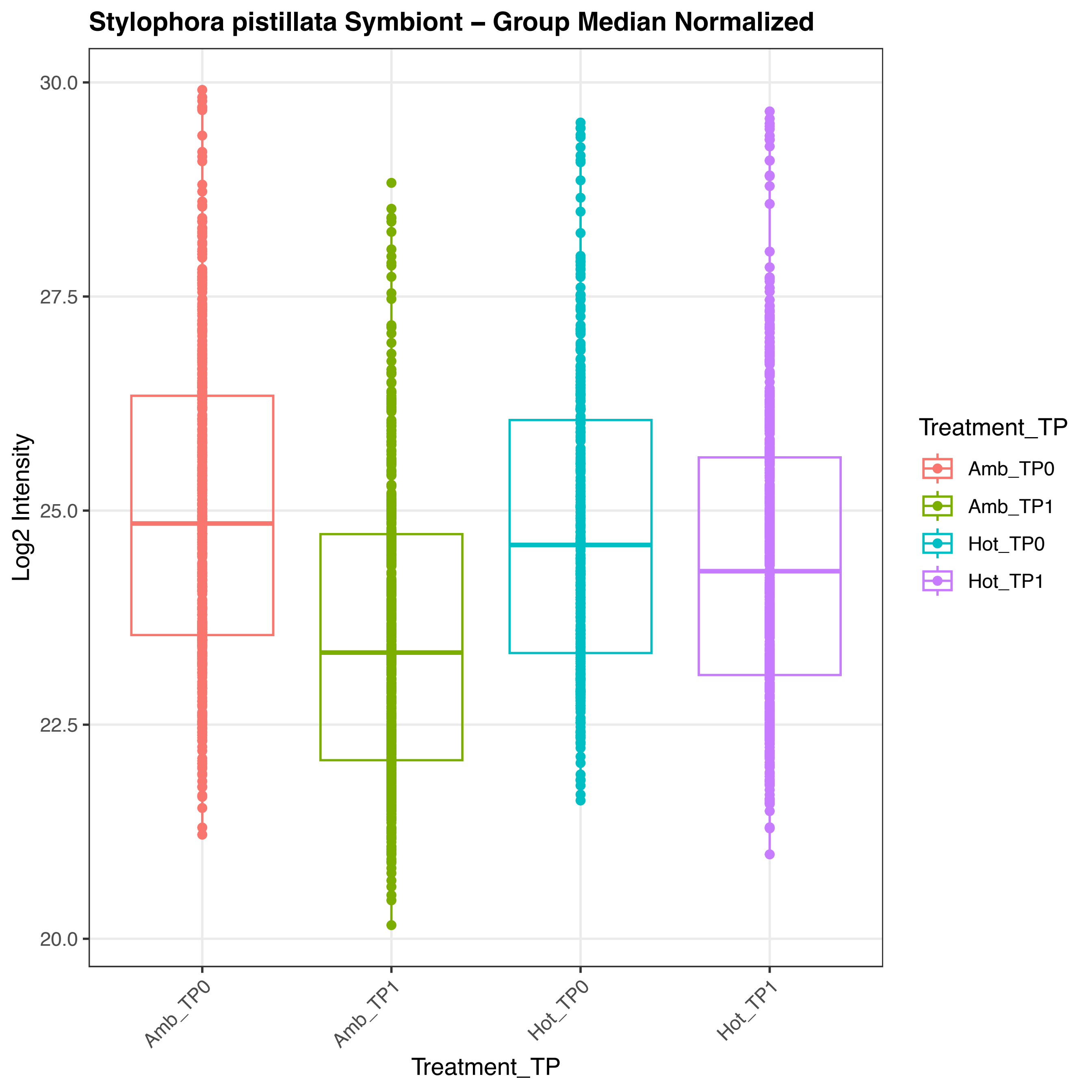

Stylophora pistillata Symbiont – Group Median Normalized
Log2 Intensity
30.0
27.5
25.0
22.5
20.0
Amb_TP0
Amb_TP1
Hot_TP0
Hot_TP1
Treatment_TP
Treatment_TP
Amb_TP0
Amb_TP1
Hot_TP0
Hot_TP1

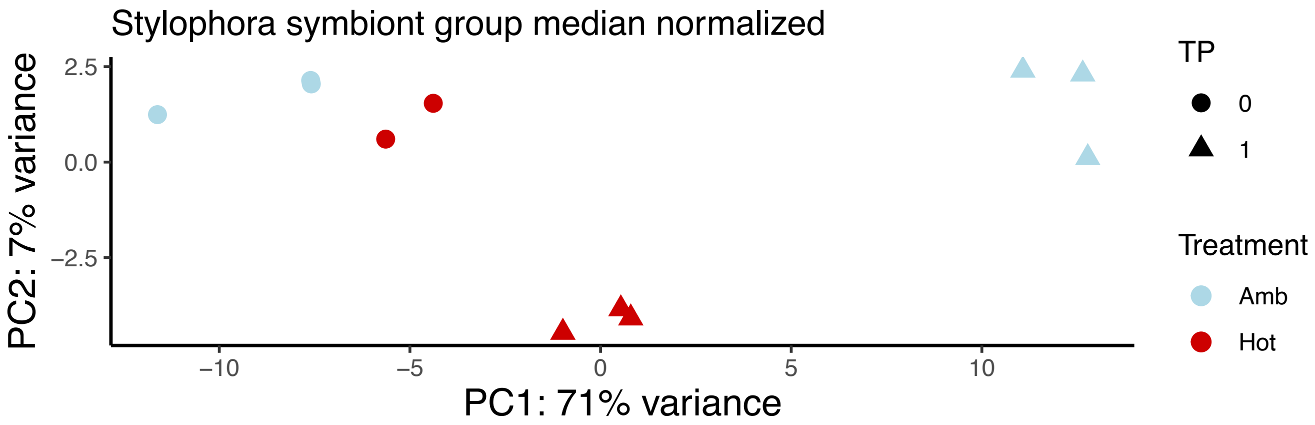

Stylophora symbiont group median normalized
PC2: 7% variance
2.5
0.0
−2.5
−10
−5
0
5
10
PC1: 71% variance
TP
0
1
Treatment
Amb
Hot
